# Identifying *RBBP7* as a Promising Diagnostic Biomarker for BK Virus-Associated Nephropathy

**DOI:** 10.1155/2022/6934744

**Published:** 2022-07-31

**Authors:** Yicun Wang, Yuxuan Wang, Di Zhang, Hao Zhang, Wei Wang, Xiaopeng Hu

**Affiliations:** ^1^Department of Urology, Beijing Chao-Yang Hospital, Capital Medical University, Beijing 100020, China; ^2^Institute of Urology, Capital Medical University, Beijing 100020, China

## Abstract

BK virus-associated nephropathy (BKVN) remains a major infectious complication due to powerful immunosuppression in kidney transplant recipients, and its histologic appearance can mimic rejection, leading to diagnostic and treatment dilemmas thus molecular diagnostic methods would be beneficial. We collected gene expression profiles of 169 kidney biopsies taken from BKVN, rejection, and stable functioning allografts, based on single sample gene set enrichment analysis and random forest algorithm, and three hallmark activities associated with DNA damage and proliferation were found to be relatively specific in BKVN. Subsequently, weighted gene co-expression network analysis and support vector machines (SVM) algorithm identified *RBBP7* as a robust and promising biomarker with high accuracy in both training and validation cohorts (AUC =0.938, 0.977, respectively). Besides, potential drugs for BKVN treatment such as mepacrine were discovered, which may contribute to targeted antiviral therapy and effective patient management rather than simply reducing the doses of immunosuppressive agents in clinical practice. *RBBP7* (retinoblastoma binding protein 7) serves as component of serval complexes that regulate chromatin metabolism and functions in affecting DNA replication and controlling cell proliferation. In this research, upregulation of *RBBP7* was found to be associated with the higher infiltration of CD8 naïve T, iTreg, and neutrophil cells and the lower amounts of Th1, central memory T, NKT, CD8 T, and dendritic cells. Moreover, the infiltration of Th1, Th17, and NKT cells was steadily different between BKVN and rejection allografts through immune cell assessment. In conclusion, we identified and verified *RBBP7* as a molecular biomarker for BKVN diagnosis, which demonstrated great distinguishing ability with allograft rejection and would support clinical decision-making.

## 1. Introduction

Nowadays, BK virus-associated nephropathy (BKVN) remains a thorny problem for renal transplant patients due to powerful immunosuppressive protocols after renal transplantation [1]. Usually occurring in the first year after transplantation when immunosuppression reaches the highest, BKVN leads to graft dysfunction in more than 90% and graft loss in over 50% of the affected individuals [2–4]. Under strong immunosuppression, latent BK virus (BKV) reactivates in the allografts, leading to inflammatory stromal nephropathy associated with functional impairment [5]. Even if both BKVN and rejection are prominent causes of kidney damage and may eventually result in graft loss, BKVN causes worse renal function 3 years after diagnosis than rejection [6]. Therefore, a timely and accurate diagnosis of BKVN can be beneficial to prompt treatment and preserve renal function thus improving prognosis for kidney transplant patients.

At present, the diagnosis of BKVN mainly depends on clinical experience and histological evidence of viral cytopathic changes in tubular epithelial cells accompanied by typical viral inclusions, which are positive for SV40T antigen in allograft biopsies [7]. However, the focal and heterogeneous natures of early BKVN easily lead to false-negative biopsy results [5]. Moreover, BKVN is histologically characterized by a plasma-cell and lymphocytic-rich interstitial nephritis, which is difficult to be distinguished from acute rejection (AR) allografts [8]. Clinically, these two diseases are treated in exactly opposite ways thus further highlighting the necessity for accurate diagnosis [8, 9]. Supplementing empirical histologic classifications with data-driven molecular estimates of disease states offers many advantages; for instance, the molecular measurements are objective, highly reproducible, and require relatively small amounts of tissue [10]. Thus, robust molecular biomarkers would be beneficial to BKVN diagnosis in clinical practice.

Since no specific antiviral therapies have been developed, reducing the doses of immunosuppressive agents remains the most common and effective therapy of BKVN [11]. Nevertheless, it may lead to allograft rejection and the leading cause of allograft loss was rejection after immunosuppression reduction instead of uncontrolled viral infection in nearly half of patients after BKVN [11], so targeted therapeutic strategies are conducive to avoiding this issue. In our study, two advanced and widely accepted algorithms, random forest (RF) and support vector machine-recursive feature elimination (SVM-RFE), were used to select biomarkers with robust and satisfying performance for BKVN diagnosis. Moreover, several potential drugs were discovered based on the Connectivity Map (CMap) database, which might provide novel approaches for clinical treatment of BKVN in renal transplant patients.

## 2. Materials and Methods

### 2.1. Data Collection and Preprocessing

After systematically screening the Gene Expression Omnibus (GEO) and ArrayExpress database, two microarray datasets (GSE75693 [12] and GSE72925 [13]), which simultaneously contained gene expression profiles of kidney allograft biopsies with histologically confirmed BKVN and rejection, were included for analysis. To be specific, the dataset GSE75693 consisted of 15 BKVN, 15 AR, and 30 stable functioning (STA) samples, while the dataset GSE72925 was comprised of 10 BKVN, 26 T-cell-mediated rejection (TCMR), and 73 STA samples. The above two datasets were both based on GPL570 platform (Affymetrix Human Genome U133 Plus 2.0 Array) and they were utilized as training and validation cohorts, respectively. The detailed demographic information can be found in Supplementary Table 1. Before analysis, data normalization was conducted through “limma” package then log_2_ transformation was performed [14].

### 2.2. Study Design

**Figure 1:**
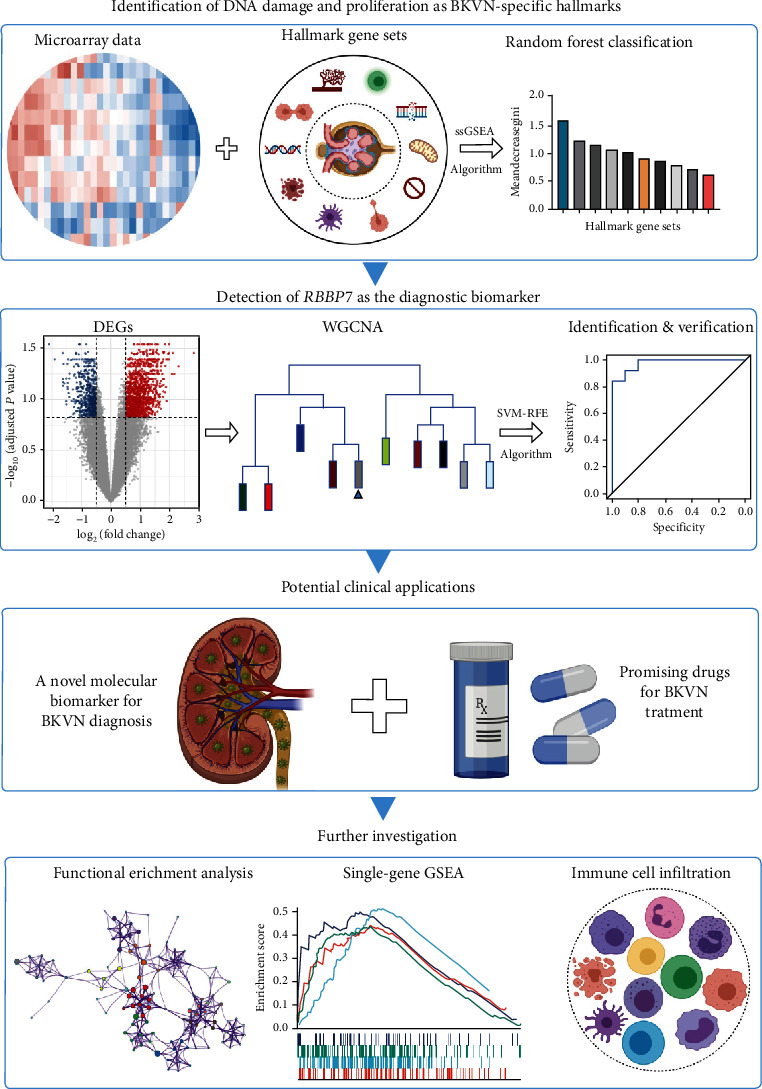
The main structure of the current study. BKVN: BK virus-associated nephropathy. ssGSEA: single sample gene set enrichment analysis. DEGs: differentially expressed genes. WGCNA: weighted gene co-expression network analysis. SVM-RFE: support vector machine-recursive feature elimination. GSEA: gene set enrichment analysis.

As illustrated in Figure 1, we firstly assessed the performances of 50 hallmark biological states or processes in three types of graft tissues by applying single sample gene set enrichment analysis (ssGSEA) algorithm, then the RF classifier approach was used to screen BKVN-specific hallmarks. Meanwhile, differential gene expression analysis was performed to identify BKVN-associated differentially expressed genes (DEGs), then a scale-free co-expression network was constructed and the module most correlated with BKVN-specific hallmarks was identified; genes included in the corresponding module were defined as BKVN-specific genes. Above candidate genes were further employed in SVM-RFE to select diagnostic genes with robust and promising values, which were also verified in external validation cohort. Besides, we applied CMap database to discover potential drugs for BKVN treatment. In the process of further investigation, functional enrichment and immune cell analyses were performed to explore underlying gene functions and specific immune cell changes in the pathogenesis of BKVN.

### 2.3. Hallmark Activities Selection in BKVN

In Molecular Signatures Database (MSigDB), we collected 50 hallmark gene sets, which summarize and represent well-defined biological states or processes with minimal overlap or redundancy. The enrichment levels of hallmark activities were quantified using the ssGSEA algorithm in each sample based on the transcriptome profiling data and corresponding gene sets derived from MSigDB, and then the enrichment-score matrix underwent z-score normalization for display. Subsequently, we utilized RF algorithm, a supervised classification method based on an ensemble of decision trees, to estimate the importance of hallmarks in distinguishing BKVN samples. Feature importance corresponded to the Gini importance measure was used to rank hallmarks in the RF classifier by “randomForest” R package [15].

### 2.4. Identification and Functional Enrichment Analysis of DEGs in BKVN

We conducted differential analyses to identify common DEGs in BKVN in comparison with AR and STA samples through “limma” R package [14]. The threshold was set at the absolute value of log_2_-fold change >0.5 and the adjusted *P* value <0.15; candidate DEGs were selected for further analysis. Moreover, we used Metascape (https://metascape.org/gp/index.html#/main/step1) to perform functional enrichment analysis with the threshold of *P* value =0.01, Minimum Overlap =3, and Minimum Enrichment =1.5 [16].

### 2.5. Screening of BKVN-Specific Genes

Weighted Gene Co-expression Network Analysis (WGCNA) is a capable approach widely employed to translate expression data into co-expression modules and investigate relationships between modules and phenotypic traits. By using WGCNA, genes with similar patterns were clustered based on expression profiles of DEGs in BKVN samples through “WGCNA” R package [17]. The module most positively correlated with BKVN-specific hallmarks was regarded as the key module and genes involved in it were defined as BKVN-specific genes. Mean values of gene significance across modules were also calculated to further confirm the role of key module.

### 2.6. Discovery of Novel Therapeutics

Predicated on the CMap database (https://portals.broadinstitute.org/cmap/), we probed into the potential compounds in BKVN treatment [18]. This database has been broadly employed for drug discovery based on integrative analyses of gene expression profiles and drug-related signatures [19, 20]. Drugs with similar or opposite genetic changes to BKVN-specific genes were filtrated for further discussion.

### 2.7. Selection and Validation of the Diagnostic Gene

SVM-RFE, an efficient feature selection algorithm in eliminating the overfitting of data, combines linear support vector machines (SVM) with feature selection backward elimination [21]. In this research, we exploited SVM-RFE to select robust genes for distinguishing BKVN from AR and STA samples, and intersected top-ranked genes were finally considered diagnostic genes, whose diagnostic values were verified in the validation set GSE72925. Receiver-operating characteristic (ROC) curves were employed to measure the diagnostic performance, and area under the ROC curve (AUC) was calculated by “pROC” R package [22]. Besides, genes previously reported for intragraft diagnosis of BKVN [13, 23] were selected and their diagnostic values were compared with that of genes identified in our research. Furthermore, we conducted gene set enrichment analysis (GSEA) of diagnostic genes to validate and explore their underlying roles during the pathogenesis of BKVN.

### 2.8. Evaluation of Immune Cells

To quantify and compare the amounts of immune cells in three types of allograft statuses, ImmuCellAI (http://bioinfo.life.hust.edu.cn/ImmuCellAI#!/) was applied, which owns the unique advantage of accurately estimating the abundance of T-cell subsets [24]. Besides, the correlations between infiltration of immune cells and expression levels of diagnostic genes were also assessed.

## 3. Results

### 3.1. Data Series Screening and Study Design

After systematically screening, we acquired two eligible kidney transplantation cohorts containing both BKVN and rejection specimens from GEO database. More detailed information can be found above in the “Materials and Methods” section. We aimed at discovering and validating novel molecular biomarkers for BKVN diagnosis; therapeutics detection and immune characterization were also performed to deepen our understanding of the treatment and potential mechanisms of BKVN.

### 3.2. Detection of Hallmark Activities in BKVN

**Figure 2:**
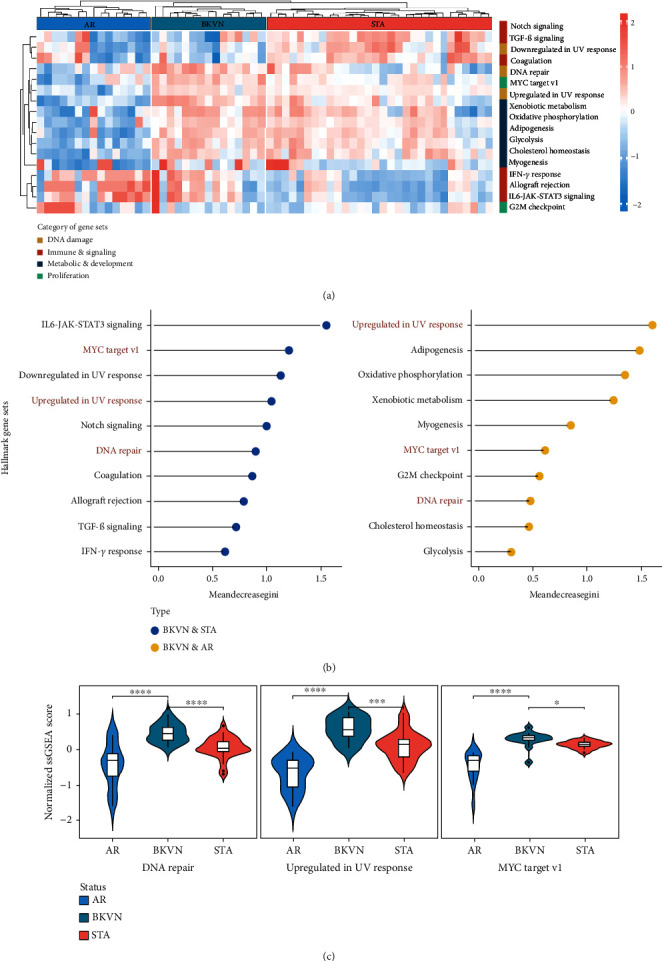
Screening of hallmark activities for BKVN. (a) Heat map of the ssGSEA scores for representative hallmark activities among AR, BKVN, and STA samples. (b) Lollipop chart shows top-ranked 10 hallmarks in BKVN vs STA (left) and BKVN vs AR (right) ordered by Gini importance through RF algorithm. (c) Violin diagram exhibits significant differences in BKVN-specific hallmarks among AR, BKVN, and STA samples. AR: acute rejection. STA: stable. ∗*P* < 0.05; ∗∗∗*P* < 0.001; ∗∗∗∗*P* < 0.0001.

We obtained the quantitative enrichment levels of 50 hallmark gene sets for each sample in GSE75693 by ssGSEA (Figure 2(a)). The results illustrated that immune-related responses or signaling, such as TGF-*β* signaling and IFN-*γ* response, differed between BKVN and STA samples, whereas metabolic and development-related gene sets differed between BKVN and AR samples. Subsequently, the RF algorithm was performed to screen significant hallmarks in distinguishing BKVN samples; the results exhibited top 10 gene sets in each classification (Figure 2(b), Supplementary Table 2). We found that three hallmark gene sets, including “MYC target v1,” “DNA repair,” and “Upregulated in UV response,” were the common part of both classifications serving as BKVN-specific hallmarks. The above three hallmarks were categorized into DNA damage and proliferation processes and could better reflect the characteristics of BKVN. As shown in Figure 2(c), those three hallmarks were significantly higher activated in BKVN samples, especially in comparison with AR or TCMR samples, which further proved that they were more suitable to reflect specific activities that occurred in BKVN thus distinguishing from rejection allografts.

### 3.3. Identification and Functional Enrichment Analysis of DEGs in BKVN

**Figure 3:**
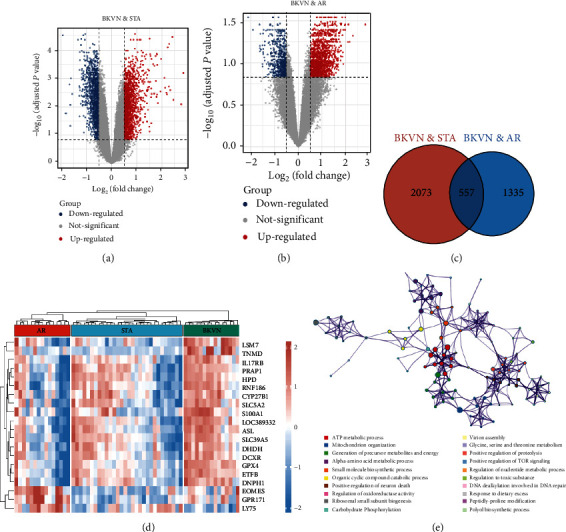
Identification and functional enrichment analysis of DEGs. (a and b) Volcano plot shows DEGs in BKVN samples compared with STA and AR samples, respectively. Blue dots denote downregulated genes, grey dots denote genes without significant differences, and red dots denote upregulated genes. (c) Venn diagram for common DEGs; the red and blue circles represent DEGs in BKVN samples contrasted with STA and AR samples, severally. (d) Heat map for the expression levels of 20 top-ranked DEGs in AR, STA, and BKVN samples. (e) Network of top 20 enriched clusters, where each node represents one statistically significant term and terms with similarity of more than 0.3 are connected by edges.

In the training cohort, a total of 2630 DEGs (1360 upregulated and 1270 downregulated) were identified between BKVN and STA samples, while 1892 DEGs (1249 upregulated and 643 downregulated) were identified between BKVN and AR samples (Figures 3(a) and 3(b)). Finally, a total of 557 common DEGs were regarded as BKVN-associated DEGs (Figure 3(c), Supplementary Table 3) and 20 top-ranked DEGs are shown in Figure 3(d). Furthermore, enrichment analysis indicated the top 20 clusters of enriched biological processes (Figure 3(e)). Those DEGs were found to be enriched in “Virion Assembly,” “DNA dealkylation involved in DNA repair,” and some other pathways, which reflected the pathogenic process of BKVN.

### 3.4. Recognition of BKVN-Specific Genes

**Figure 4:**
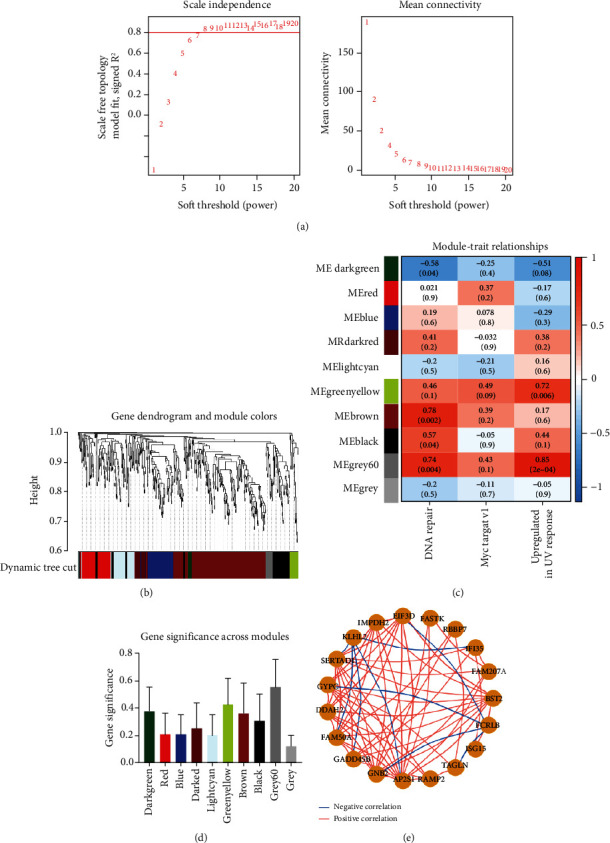
Identification of the key module by WGCNA. (a) Analysis of the scale-free fit index (left) and the mean connectivity (right) for various soft-thresholding power values. (b) Clustering dendrogram of DEGs with assigned module colors. (c) Heat map of the correlation between modules and hallmark activities of BKVN. Each cell contains the Pearson correlation coefficient and *P* value. (d) Histogram of the gene significance across modules for three hallmarks in BKVN. (e) Correlations of the expression levels of 19 genes that belong to grey60 module. Blue curves indicate negative correlations, and red curves indicate positive correlations.

To select genes functioning in BKVN-specific hallmark activities, we performed WGCNA based on expression levels of 557 DEGs in BKVN samples. As a result, ten co-expression modules were eventually identified with the following parameters: best soft-thresholding power =8, minModulesize =10, deepSplit =3, MEDissThres =0.35 (Figures 4(a) and 4(b)). According to the heat map of module-trait relationships, grey60 module had the highest correlation with three BKVN-specific hallmarks (Figure 4(c)). On the other hand, we calculated gene significance across modules for the three traits and grey60 module showed the highest significance (Figure 4(d)), which further verified that 19 genes included in the grey60 module had the highest correspondence with BKVN. Furthermore, correlation analysis also indicated tight relationships among 19 genes (Figure 4(e)).

### 3.5. Discovery of Potential Drugs for BKVN

Through the online website CMap, the top 10 drugs with similar or opposite expression patterns to BKVN were screened and displayed (Table 1). Among them, the expression changes caused by 6 drugs, including irinotecan, depudecin, camptothecin, staurosporine, doxorubicin, and mycophenolic acid, were similar to that during BKVN. In contrast, mepacrine, rifabutin, emetine, and thapsigargin showed opposite expression patterns suggesting that they might be able to reverse genetic changes during the pathogenesis of BKVN and serve as potential drugs for BKVN treatment.

### 3.6. Selection and Validation of the Diagnostic Gene

**Figure 5:**
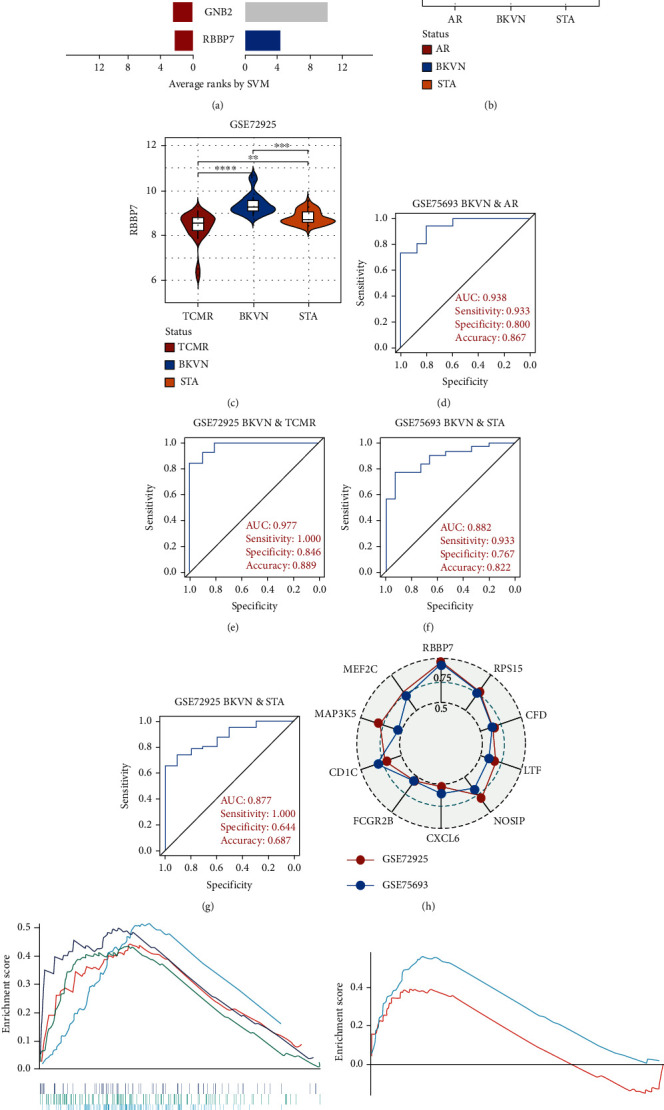
Selection and validation of the diagnostic gene. (a) Horizontal bar chart shows genes ranked by SVM-RFE. Red bars and blue bars represent the top-ranked 5 genes in comparison to AR and STA samples, respectively. (b and c) Violin plots show differences in expression levels of *RBBP7* among three types of allografts in the training and validation cohorts. (d and e) ROC curves show the diagnostic ability of *RBBP7* between BKVN and rejection samples in the training and validation cohorts. (f and g) ROC curves show the diagnostic ability of *RBBP7* between BKVN and STA samples in the training and validation cohorts. (h) Radar chart shows the AUC of *RBBP7* and other previously reported genes for BKVN diagnosis in training and validation cohorts. (i and j) GSEA reflects pathways enriched in BKVN samples with higher expression levels of *RBBP7* in training and validation cohorts. TCMR: T cell-mediated rejection. ROC: receiver-operating characteristic. AUC: area under the curve. ∗∗*P* < 0.01; ∗∗∗*P* < 0.001; ∗∗∗∗*P* < 0.0001, ns: not statistically significant.

SVM-RFE was applied for gene selection with the advantage of eliminating the overfitting of data. The top-ranked 5 genes by SVM-RFE in distinguishing BKVN and AR were *RBBP7*, *GNB2*, *GADD45B*, *FAM207A*, and *IMPDH2*. In the same way, *FCRLB*, *GYPC*, *RBBP7*, *AP2S1*, and *EIF3D* were found to be the top-ranked 5 genes in discriminating BKVN and STA (Figure 5(a), Supplementary Table 4). As a result, *RBBP7* was the only common gene with significant ability in classifying BKVN from the other two sample types. To verify the diagnostic ability of *RBBP7*, GSE72925 was employed as an external validation set. There were significant differences in the expression levels of *RBBP7* among three types of specimens (Figures 5(b) and 5(c)). In the training cohort, *RBBP7* indicated a great diagnostic power (AUC =0.938) with a specificity of 80.0% and a sensitivity of 93.3% in differentiating BKVN and AR samples (Figure 5(d)). Comparably, *RBBP7* showed even better diagnostic ability (AUC =0.977, specificity =0.846, sensitivity =1.000) in discriminating BKVN and TCMR in validation cohort (Figure 5(e)). When it comes to the contrast of BKVN and STA samples, *RBBP7* also demonstrated a reliable diagnostic accuracy (AUC =0.882) with a specificity of 76.7% and a sensitivity of 93.3% in the training cohort (Figure 5(f)), and in validation cohort, the AUC was 0.877 with 100% sensitivity and 64.4% specificity (Figure 5(g)).

Furthermore, genes for BKVN diagnosis reported in previous studies were selected, and we compared their diagnostic values with *RBBP7*. As illustrated in Figure 5(h), *RBBP7* revealed the most robust diagnostic capabilities distinctly. After that, we applied single-gene GSEA for *RBBP7* in BKVN samples. The result showed that in both training and validation cohorts, it was mainly enriched in “Upregulated in UV response” hallmark pathway (Figures 5(i) and 5(j)), confirming the previous conclusion. Besides, “Fc gamma R-mediated phagocytosis,” serving to remove antibody-opsonized antigens from systemic circulation, such as dengue virus [25], was also found to be enriched in BKVN tissues with higher expression levels of *RBBP7*. Therefore, our results ascertained the reliability of *RBBP7* from a functional perspective and explored possible pathogenic mechanisms.

### 3.7. Inference of Immune Cells in BKVN

**Figure 6:**
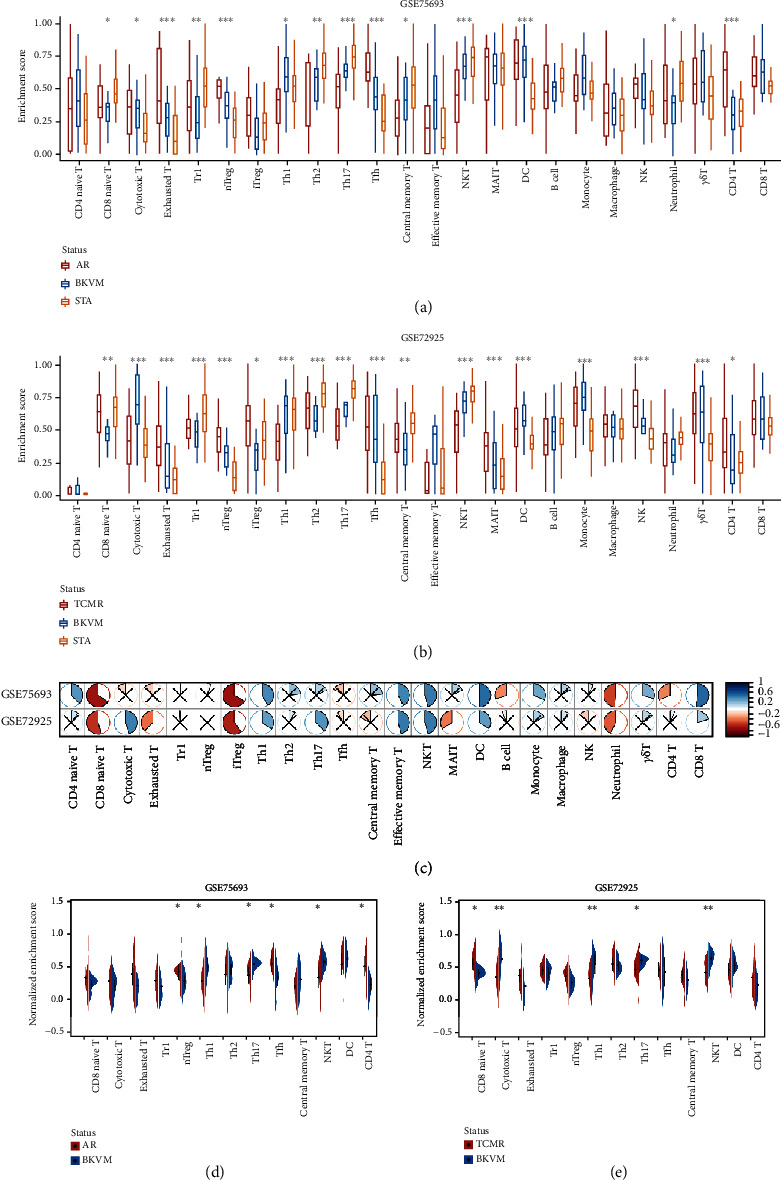
Estimation and comparison of immune cell infiltration. (a and b) Box plots show amounts of various immune cells among BKVN, AR (TCMR), and STA samples in training and validation cohorts. (c) Correlation heat map between the expression levels of *RBBP7* and immune cell infiltration in the training and validation cohorts. The pie graphs are filled in proportion to Spearman's coefficient values. Anticlockwise for negative correlations (in red), clockwise for positive correlations (in blue); the red crosses represent no statistically significant correlations (*P* > 0.05). (d and e) Half-violin plots show normalized amounts of immune cells between BKVN and rejection samples in training and validation cohorts. Immune cells with significantly different infiltration among three types of samples were incorporated. ∗*P* < 0.05; ∗∗*P* < 0.01; ∗∗∗*P* < 0.001.

By comparing the infiltration of immune cells among BKVN, STA, and AR samples (Figures 6(a) and 6(b)) and calculating correlations between immune cells and *RBBP7* (Figure 6(c)), we found that the infiltration levels of some immune cells were significantly different. Upregulation of *RBBP7* may be associated with increased levels of CD8 naive cells, iTreg cells, and neutrophil cells and decreased levels of Th1 cells, central memory T cells, NKT cells, DC cells, and CD8 T cells. After that, we compared the amounts of immune cells between BKVN and AR (TCMR), two easily confounded allograft states, separately (Figures 6(d) and 6(e)). As a result, the amounts of Th1, Th17, and NKT cells were significantly different in both training and validation cohorts.

## 4. Discussion

BKVN remained a tough issue after kidney transplantation which was seen in 1%-10% of kidney transplant recipients [2, 26], and graft loss occurred in 15%–50% of BKVN cases [27]. Although BKVN and AR are both the main causes of graft damage, clinical therapies are opposite for these two states in renal allografts [9]. For this reason, differentiating BKVN from AR is crucial in clinical practice but difficult in pathological diagnosis. Hence, intragraft molecular diagnosis may afford opportunities for increased precision to address the limitations of conventional histologic methods.

In the present study, we applied ssGSEA to estimate the enrichment levels of hallmark activities in each sample. Compared with STA samples, some immune-related pathways showed respectable discrimination, such as “IL6-JAK-STAT3 signaling,” “IFN-*γ* response,” and “TGF-*β* signaling.” However, these pathways performed poorly in discriminating between BKVN and AR, while some hallmarks associated with viral infection, such as “Upregulated in UV response,” presented great resolving abilities, further demonstrating the specificity of virus-associated processes during the pathogenesis of BKVN, especially in comparison with rejection states. As a result, three hallmark activities were detected to be relative BKVN-specific hallmarks, namely, “MYC target v1,” “DNA repair,” and “Upregulated in UV response.” Among them, “MYC target v1” was categorized into proliferation process. MYC, a transcriptional regulator overexpressed in various cancers, appears to play a direct role in preventing immune cells from effectively attacking tumor cells [28]. It has been reported that “MYC target v1” was highly enriched during human T-cell lymphotropic virus type 1 (HTLV-1) and bovine leukemia virus (BLV) infections to promote viral malignancy through enhancing cell proliferation [29]. Our results also showed an increased level of this activity in BKVN samples, which indicated that it may take part in viral spread through enhanced immunosuppression and cell proliferation in BKVN. “DNA repair” and “Upregulated in UV response” were both parts of DNA damage process. Activation of DNA damage response and recruitment of DNA repair proteins were found to be used by different viruses to manipulate the transcription and translation of host DNA [30]. As a double-stranded DNA virus, BKV would integrate its DNA into the host and replicate in large quantities by these two biological processes during BKVN progression. In brief, proliferation and DNA damage processes may be specific in virus infection compared with rejection, which are more suitable for distinguishing BKVN samples, instead of immune responses. Similarly, in a recent study, researchers found that polyomavirus 5-gene set reliably distinguished BKVN from TCMR, but the other two immune-related gene sets demonstrated suboptimal diagnostic performances [23], further illustrating virus-associated biological processes are more representative for BKVN.

Based on WGCNA, a 19-gene module was found to be closely related to BKVN-specific hallmark activities as described previously, and these 19 genes were tightly linked with each other. Among them, SVM-RFE selected *RBBP7* as the most promising gene for BKVN diagnosis, especially in distinguishing from rejection samples with an AUC of 0.938 and 0.977 in the training and validation cohorts, respectively. Moreover, *RBBP7* revealed an excellent and robust diagnostic ability, far superior to other diagnostic genes proposed in previous studies. *RBBP7* (retinoblastoma binding protein 7, chromatin remodeling factor) is a ubiquitously expressed nuclear protein that was found in many histone deacetylase (HDAC) complexes [31]. It plays an important role in transcription and chromatin assembly and is involved in many biological processes including viral life cycle, which is followed by all viruses to ensure survival [32]. This process included several steps, such as attachment and entry of the virus particle, decoding of genome information and assembly, and release of viral particles containing the genome [32]. To date, studies have reported the role of *RBBP7* in several diseases, such as Alzheimer's disease [33] and Wilms Tumor 1 [34], while our results of single-gene GSEA showed that “Fc gamma R-mediated phagocytosis” pathway was significantly enriched in BKVN samples with higher expression levels of *RBBP7*, which set the foundation for researches of *RBBP7* in virus-associated diseases, also in BKVN. After being triggered by clustering of Fc gamma R at sites where leukocytes are bound to the opsonized particles, neutrophils and macrophages engulf IgG-coated particles, which is an essential part of the innate immune response [35]. Recently, it was found to be significantly enriched in human lung epithelial cells infected with SARS-CoV-2 [36]. These findings indicate that *RBBP7* may exert its effect in BKVN through this pathway, which needs further experimental verification.

Currently, the treatment of BKVN mainly relies on reducing immunosuppression [37], which may increase the incidence of allograft rejection. Therefore, we utilized CMap database to explore potential drugs for BKVN treatment. Consequently, drugs leading to opposite expression alterations with BKVN deserved more attention. Among them, it is remarkable that mepacrine with an effect of restraining virus replication was identified as a potent Ebola virus inhibitor both *in vitro* and *in vivo* and showed an anti-SARS-CoV-2 activity [38]. Therefore, these drugs exhibited potential in treating BKVN and may protect patients from side effects of reducing immunosuppressive in clinical practice. However, due to its limitation of online results, further experimental and clinical practices were needed to prove their roles.

As a disease caused by virus infection, changes of immune cell infiltration during disease development are nonnegligible. Accordingly, we estimated the amounts of immune cells, especially T cell subsets in three types of allograft statuses and tried to detect their differences. Compared with AR, the amounts of nTreg, Th1, Th17, Tfh, NKT, and CD4^+^ T cells exhibited markable differences in BKVN. While CD8^+^ naïve, cytotoxic T, Th1, Th17, and NKT cells indicated significant differences between BKVN and TCMR samples. The above results were not the same, probably due to inherent differences existing between AR and TCMR samples. Therefore, we mainly focused on three types of immune cells with similar changes. Deriving from naive CD4^+^ T helper cells, Th1 cells express transcription factor T-bet and produce IFN-*γ*, which can control virus spread and contribute to the production and maintenance of cytotoxic T cells [39, 40]. As a subset of T helper cells, Th17 cell signaling pathway leads to the secretion of proinflammatory cytokines such as IL-17A, which is important for host defense against fungal and extracellular bacterial infections. Researchers reported that the infection of attenuated rabies virus increased the infiltration of Th1 cells and Th17 cells [41], which was consistent with our results. NK and NKT cells constitute a significant proportion of liver-infiltrating lymphocytes during HCV infection and promote virus-specific adaptive responses [42]. In our study, the amounts of NKT cells in BKVN samples were incremental, suggesting that they may act in similar manners in BKVN. Overall, Th1, Th17, and NKT cells showed differences between BKVN and rejection allografts, suggesting that they may play significant roles in BKVN. In accordance with correlations between the expression level of *RBBP7* and amounts of immune cells, we found that *RBBP7* was negatively correlated with iTreg cells that play an immune-suppressing role, while positively correlated with Th1 cells, central memory T cells, NKT cells, and CD8^+^ T cells. Therefore, we speculate that higher levels of *RBBP7* expression may enhance T cell-mediated immune responses or RBBP7 would be stimulated during the activation of these immune cells, which deserves further verification.

In this study, using BKVN-specific hallmarks, *RBBP7* with powerful and stable diagnostic ability was identified and verified, which provides a potential solution for clinical difficulty in distinguishing BKVN from rejection, especially TCMR. What's more, some potential therapeutic agents for BKVN were detected, which provided new ideas for the treatment of BKVN and may reduce the side effects of current treatment—diminishing the dose of immunosuppressive drugs. However, there are still some limitations to this study. Sample sizes of the datasets we included were insufficient, and a larger cohort was needed to verify the diagnostic ability of *RBBP7*. Potential drugs and mechanisms proposed in this study lack direct evidence and require further experimental verification.

## 5. Conclusions

We identified and verified *RBBP7* as a molecular biomarker for BKVN diagnosis, which demonstrated great distinguishing ability, and it was beneficial for clinical decision-making. Additionally, our exploration of potential therapeutic drugs may provide insights for the development of novel therapeutics and possibly break out the current awkward situation in BKVN treatment.

## Figures and Tables

**Table 1:** Results of CMap analysis.

Rank	Cmap name	Mean	*N*	Enrichment	*P*-value	Description
1	Irinotecan	0.938	3	0.997	0.00001	Topoisomerase inhibitor
2	Depudecin	0.831	2	0.967	0.00183	HDAC inhibitor
3	Camptothecin	0.851	3	0.929	0.00066	Topoisomerase inhibitor
4	Staurosporine	0.756	4	0.885	0.00018	PKC inhibitor
5	Mepacrine	-0.729	2	-0.879	0.02956	Cytokine production inhibitor
6	Doxorubicin	0.737	3	0.854	0.00593	Topoisomerase inhibitor
7	Rifabutin	-0.692	3	-0.848	0.00697	Protein synthesis inhibitor
8	Emetine	-0.685	4	-0.844	0.00105	Protein synthesis inhibitor
9	Thapsigargin	-0.652	3	-0.838	0.00853	ATPase inhibitor
10	Mycophenolic acid	0.711	3	0.834	0.00929	Dehydrogenase inhibitor

## Data Availability

Two GEO datasets (GSE75693 and GSE72925) included in this research were obtained from GEO database (https://www.ncbi.nlm.nih.gov/geo/).

## Supplementary Materials

Supplementary Materials Supplementary Table 1: Demographic information of patients included in GSE72925. Supplementary Table 2: Top-ranked 10 hallmarks screened by RF algorithm. Supplementary Table 3: BKVN-associated DEGs compared with STA and AR samples. Supplementary Table 4: The average rank of 19 genes included in the grey60 module calculated by support vector machine - recursive feature elimination algorithm.
