# Supplementary material for: Identifying RBBP7 as a Promising Diagnostic Biomarker for BK Virus-Associated Nephropathy
Source: J Immunol Res. 2022 Jul 31;2022:6934744. doi: 10.1155/2022/6934744 (PMC9357817; doi:10.1155/2022/6934744)
Supplement: Supplementary Materials — Supplementary Table 1: Demographic information of patients included in GSE72925. Supplementary Table 2: Top-ranked 10 hallmarks screened by RF algorithm. Supplementary Table 3: BKVN-associated DEGs compared with STA and AR samples. Supplementary Table 4: The average rank of 19 genes included in the grey60 module calculated by support vector machine - recursive feature elimination algorithm. [file 6934744.f1.docx]

**Supplementary Materials**

**Supplementary Table 1.** Demographic information of patients included in GSE72925. BKVN: BK virus-associated nephropathy. TCMR: T cell-mediated rejection. STA: stable. NS: no statistical significance. # BKVN and TCMR samples were not statistically significant.

| Main clinical variables | BKVN | TCMR | STA | *P* value |
| --- | --- | --- | --- | --- |
| *No*. of biopsies | 10 | 26 | 73 |  |
| Mean donor age (yr±SD) | 24±10 | 30±11 | 31±11 | NS |
| Mean recipient age (yr±SD) | 14±4 | 11±6 | 11±6 | NS |
| Recipient gender (% Female) | 20 | 21 | 36 | NS |
| % prior history of acute rejection | 15 | 10 | 4 | NS |
| % Steroid-free maintenance immunosuppression | 60 | 58 | 70 | NS |
| CNI-based (%) | 100 | 100 | 100 | NS |
| Type of transplant (% living donor) | 66 | 71 | 70 | NS |
| Time post-transplant (mo±SD) | 10±9 | 11±7 | 13±19 | NS |
| Type of Biopsy (% Cause) | 60 | 21 | 0 | ＜0.0001 |
| Serum creatinine at the timeof biopsy (mg/dL) | 1.48±0.56 | 1.84±0.50 | 0.72±0.36 | ＜0.0001 |
| Kidney histology lesions (Acute Banff score for each renal compartment; 0-3) (mean±sd; range) | | | | |
| Acute tubuli (t) | 1.7±1 (1-3) | 1.5±0.9 (1-3) | 3±0.18 (0-1) | # |
| Acute Interstitium (i) | 1.5±1.5 (1-2) | 2.2±0.9 (1-3) | 0 | # |
| Acute glomeruli (ag) | 0.1±0.3 (0-1) | 0.2±0.3 (0-1) | 0 | # |
| Acute vascular (av) | 0 | 0 | 0 | NS |

**Supplementary Table 2.** Top-ranked 10 hallmarks screened by RF algorithm. RF: random forest. AR: acute rejection; STA: stable functioning.

| BKVN vs STA | | | |
| --- | --- | --- | --- |
| Hallmark gene sets | Category | Description | MeanDecreaseGini |
| IL6_JAK_STAT3_SIGNALING | immune | IL6 STAT3 signaling during acute phase response | 1.559320071 |
| MYC_TARGETS_V1 | proliferation | MYC targets variant 1 | 1.213785043 |
| UV_RESPONSE_DOWN | DNA damage | UV response: down-regulated genes | 1.135709302 |
| UV_RESPONSE_UP | DNA damage | UV response: up-regulated genes | 1.053444257 |
| NOTCH_SIGNALING | signaling | Notch signaling | 1.005445623 |
| DNA_REPAIR | DNA damage | DNA repair | 0.900141001 |
| COAGULATION | immune | Coagulation cascade | 0.868307344 |
| ALLOGRAFT_REJECTION | immune | Allograft rejection | 0.791104964 |
| TGF_BETA_SIGNALING | signaling | TGF beta signaling | 0.721164635 |
| INTERFERON_GAMMA_RESPONSE | immune | Interferon gamma response | 0.613922403 |
| BKVN vs AR | | | |
| Hallmark gene sets | Category | Description | MeanDecreaseGini |
| UV_RESPONSE_UP | DNA damage | UV response: up-regulated genes | 1.848852909 |
| ADIPOGENESIS | development | Adipocyte development | 1.715850977 |
| OXIDATIVE_PHOSPHORYLATION | metabolic | Oxidative phosphorylation and citric acid cycle | 1.557831886 |
| XENOBIOTIC_METABOLISM | metabolic | Metabolism of xenobiotics | 1.440981315 |
| MYOGENESIS | development | Muscle differentiation | 0.968942849 |
| MYC_TARGETS_V1 | proliferation | MYC targets variant 1 | 0.694921021 |
| G2M_CHECKPOINT | proliferation | Cell cycle G2/M checkpoint | 0.648890981 |
| DNA_REPAIR | DNA damage | DNA repair | 0.546569419 |
| CHOLESTEROL_HOMEOSTASIS | metabolic | Cholesterol homeostasis | 0.528286108 |
| GLYCOLYSIS | metabolic | Glycolysis and gluconeogenesis | 0.345690476 |

**Supplementary Table 3.** BKVN-associated DEGs compared with STA and AR samples. FC: fold change.

| Gene | BKVN vs STA | | BKVN vs AR | |
| --- | --- | --- | --- | --- |
|  | logFC | Adjusted *P* value | logFC | Adjusted *P* value |
| AFAP1L2 | -1.925013371 | 0.000030024 | -0.756367351 | 0.149870298 |
| FSTL3 | 1.106225027 | 0.000042791 | 0.792862381 | 0.0783784 |
| C9orf16 | 0.874385061 | 0.000042791 | 0.619323631 | 0.104673369 |
| GPX4 | 1.167926931 | 0.000042791 | 1.700065206 | 0.052362868 |
| SERINC5 | -1.33204584 | 0.000042791 | -1.078615201 | 0.028818306 |
| NTHL1 | 1.020303568 | 0.000042791 | 0.774601252 | 0.129578348 |
| TNMD | 2.287475027 | 0.000059257 | 1.80322281 | 0.062878082 |
| RAB24 | 0.930926484 | 0.000087063 | 1.14111761 | 0.083596597 |
| HNRNPAB | 0.57409476 | 0.000102262 | 0.704307739 | 0.083596597 |
| RP3-327A19.5 | -1.176809823 | 0.000104022 | -1.192915932 | 0.035072496 |
| EIF3G | 1.038884435 | 0.000116893 | 1.351277883 | 0.126225875 |
| GOLPH3 | -0.977147118 | 0.000119546 | -0.631648076 | 0.131902327 |
| LSM7 | 1.539894808 | 0.000159201 | 1.633542164 | 0.056482781 |
| PPP1R7 | 0.837231776 | 0.000159201 | 0.930125498 | 0.086877481 |
| BST2 | 1.302546673 | 0.00016494 | 1.362944829 | 0.124113253 |
| PHPT1 | 0.962063863 | 0.000168843 | 1.168757858 | 0.056751253 |
| KDM6A | -0.647807198 | 0.000177517 | -0.559309871 | 0.137831986 |
| PIN1 | 0.989478684 | 0.000194947 | 0.89569978 | 0.118698397 |
| RPS19BP1 | 1.026544273 | 0.000207089 | 1.231588222 | 0.09533455 |
| INTS2 | -0.969241031 | 0.000219267 | -0.698992789 | 0.085090795 |
| PCDHB16 | -1.253407945 | 0.000223289 | -1.159919229 | 0.076094854 |
| SCAF8 | -0.818187556 | 0.000230657 | -0.643525086 | 0.100615447 |
| TSTA3 | 1.03517571 | 0.000235106 | 1.009924218 | 0.062878082 |
| NDUFA3 | 1.170960856 | 0.000247997 | 1.189705753 | 0.137701007 |
| EFNA5 | -0.851387835 | 0.00024902 | -0.628387991 | 0.139448345 |
| ENAM | -0.770005489 | 0.000273075 | -0.605660242 | 0.081730013 |
| PRDX4 | 0.954880547 | 0.000273075 | 0.745519538 | 0.144099562 |
| S1PR1 | -0.866455333 | 0.000290818 | -0.723805499 | 0.071237241 |
| MRPL54 | 1.163104942 | 0.000315938 | 1.380349513 | 0.103698382 |
| TPRN | 0.91703682 | 0.000329524 | 0.824482706 | 0.042901667 |
| RALY | 0.72228596 | 0.000374195 | 0.86633928 | 0.133292845 |
| CD2BP2 | 0.665137622 | 0.000374195 | 0.829957969 | 0.077495668 |
| RFXANK | 0.88489599 | 0.000380149 | 1.289849126 | 0.055194593 |
| TRAPPC5 | 1.148386707 | 0.000395564 | 0.946996189 | 0.126618617 |
| ZNF236 | -0.925234453 | 0.000397692 | -0.847589731 | 0.065519916 |
| PPP1R14B | 1.124205615 | 0.00040979 | 0.998888688 | 0.1319451 |
| C5orf30 | -0.900345684 | 0.000425169 | -0.683485558 | 0.1319451 |
| MAF1 | 0.827337212 | 0.000431031 | 1.274272042 | 0.066640616 |
| C7orf73 | -0.728555807 | 0.00044233 | -0.850067316 | 0.115150183 |
| LAMTOR4 | 0.89097755 | 0.000449786 | 1.313214496 | 0.0783784 |
| NOP16 | 0.8104333 | 0.000455929 | 1.027748689 | 0.096004993 |
| ANKRD19P | 0.855242983 | 0.000478272 | 0.947507836 | 0.13774247 |
| ZNF431 | -0.643291304 | 0.000490445 | -0.63965512 | 0.111655625 |
| MINOS1P1 | -1.266430304 | 0.000497534 | -0.890408158 | 0.127501956 |
| HIVEP1 | -0.627638405 | 0.000502379 | -0.802148414 | 0.056482781 |
| UBQLN2 | -0.658585727 | 0.00051069 | -0.73688538 | 0.04039858 |
| CAMK1 | 0.840818623 | 0.000512002 | 0.763353133 | 0.122394028 |
| PRPF4B | -0.646054413 | 0.000514157 | -0.622614933 | 0.041586502 |
| NOL11 | 0.678626763 | 0.000519936 | 0.919482911 | 0.135874269 |
| RIMKLB | -0.684482096 | 0.000525492 | -0.566207784 | 0.101759329 |
| MFSD6 | -0.884785985 | 0.000525492 | -0.762687102 | 0.061086025 |
| STAP2 | 0.894773787 | 0.000537438 | 1.303676301 | 0.04696954 |
| BAI2 | 0.50939893 | 0.000554698 | 0.590992211 | 0.04039858 |
| THAP7 | 0.715107833 | 0.000559554 | 0.990762074 | 0.119055748 |
| DDIT3 | 1.034519974 | 0.000560101 | 1.091346716 | 0.109834863 |
| VSIG10 | -0.974150184 | 0.000596421 | -0.690971011 | 0.145031432 |
| NDUFA11 | 0.694743328 | 0.000597467 | 1.243288313 | 0.065264277 |
| MRPL28 | 0.873362402 | 0.000597467 | 0.970302496 | 0.133693938 |
| RAPH1 | -0.650480893 | 0.000597467 | -0.567172424 | 0.052832389 |
| KIAA1715 | -0.742125435 | 0.000613185 | -0.741343313 | 0.078897913 |
| OGFOD3 | 0.751813456 | 0.000613185 | 0.736651036 | 0.04039858 |
| ABHD17A | 0.63279945 | 0.00061792 | 0.502718812 | 0.110078572 |
| PARG | -0.881901757 | 0.000635953 | -0.617130276 | 0.135131573 |
| MPG | 0.737679214 | 0.000638693 | 1.041231044 | 0.061086025 |
| GFER | 0.646486761 | 0.00064182 | 0.797586733 | 0.061898706 |
| NOB1 | 0.761702271 | 0.000659448 | 1.021022468 | 0.075498388 |
| C6orf62 | -0.568476387 | 0.000689036 | -0.680909876 | 0.099115143 |
| SAMD8 | -0.720635871 | 0.000699843 | -0.670475358 | 0.04039858 |
| ZNF850 | -0.81871547 | 0.000718978 | -0.809961739 | 0.144099562 |
| STARD10 | 0.748366733 | 0.000718978 | 1.153939236 | 0.04039858 |
| BAD | 0.668404416 | 0.000718978 | 0.824245019 | 0.04039858 |
| ZCCHC2 | -0.63964437 | 0.000718978 | -0.746043998 | 0.041586502 |
| C6orf1 | 0.715045299 | 0.00072086 | 0.87131047 | 0.078897913 |
| HERPUD2 | -0.627397477 | 0.000737905 | -0.754953727 | 0.066687695 |
| DDX49 | 0.742748053 | 0.000737905 | 0.811374287 | 0.055598002 |
| MDM4 | -0.567551642 | 0.000742224 | -0.657698627 | 0.04039858 |
| CYHR1 | 0.503719542 | 0.000742224 | 0.712697698 | 0.043914519 |
| FAM173A | 0.944490124 | 0.000742295 | 1.023143168 | 0.060189486 |
| TESC | 0.583783245 | 0.000742295 | 0.507085776 | 0.110078572 |
| SAC3D1 | 1.101906118 | 0.000747894 | 1.003720892 | 0.081767444 |
| BRICD5 | 0.71348082 | 0.000766229 | 0.662600261 | 0.045347239 |
| AAMP | 0.61912799 | 0.000770068 | 0.672953016 | 0.081767444 |
| RBM12B | -0.608252069 | 0.000784871 | -0.873079891 | 0.104607039 |
| DEF8 | 0.591305019 | 0.000784871 | 0.80681108 | 0.044354091 |
| C19orf24 | 0.870040001 | 0.000808862 | 1.131513357 | 0.07922176 |
| NAA10 | 0.714161616 | 0.000813127 | 1.339923543 | 0.056166142 |
| C14orf166 | 0.56251482 | 0.000814801 | 0.830343611 | 0.109070527 |
| ALKBH3 | 0.613834199 | 0.00081828 | 0.831629016 | 0.138842562 |
| TIMM13 | 0.709306415 | 0.000829298 | 0.630869958 | 0.148282834 |
| LOC440934 | -1.104641719 | 0.000848361 | -1.08690076 | 0.120614999 |
| DNAJC17 | 0.827124316 | 0.000848361 | 0.99715188 | 0.058277801 |
| R3HCC1 | 0.904775854 | 0.000872597 | 1.121388144 | 0.044114539 |
| VPS4B | -0.552274916 | 0.000914805 | -0.569848929 | 0.042423702 |
| FAM207A | 0.625301193 | 0.000917993 | 0.628045798 | 0.04039858 |
| HMOX2 | 0.722938662 | 0.000920002 | 0.866115798 | 0.056166142 |
| DAPK1 | -0.705880103 | 0.000932945 | -0.590227109 | 0.066687695 |
| MRPL55 | 0.856262732 | 0.000986731 | 1.211728787 | 0.058277801 |
| SDF2L1 | 1.04931979 | 0.000991629 | 0.867564528 | 0.129777291 |
| NOP14 | -0.820085226 | 0.000991629 | -0.525094182 | 0.109519047 |
| TRIB2 | -0.682895004 | 0.000991629 | -0.662409207 | 0.064412191 |
| FLAD1 | 0.623851447 | 0.000995876 | 0.812412551 | 0.098408974 |
| DUS1L | 0.981309776 | 0.001037713 | 1.153624353 | 0.086957159 |
| DCTD | 0.546015442 | 0.001059131 | 0.525979176 | 0.098921463 |
| IFI35 | 1.158226965 | 0.001064648 | 0.955915394 | 0.145040987 |
| EDF1 | 0.855531655 | 0.001089293 | 1.653757498 | 0.112088857 |
| ZNF501 | -0.745135442 | 0.001152915 | -0.569775951 | 0.120936418 |
| SF3B5 | 0.892109436 | 0.001208767 | 1.343198583 | 0.123712395 |
| ICK | -0.591209715 | 0.001266216 | -0.599854922 | 0.13222443 |
| MAP4K2 | 0.66882466 | 0.001299215 | 0.815515378 | 0.06297727 |
| TMEM87B | -0.692782851 | 0.001301319 | -0.693715842 | 0.148100937 |
| AIP | 0.783363006 | 0.001310309 | 1.092271084 | 0.061898706 |
| CREB3 | 0.707765506 | 0.001341475 | 0.933216336 | 0.085090795 |
| RBBP7 | 0.571679041 | 0.001345249 | 0.847950344 | 0.131340034 |
| NHP2 | 0.755580514 | 0.00134555 | 1.075821041 | 0.077321195 |
| SURF6 | 0.910868107 | 0.00134794 | 1.2403727 | 0.037255148 |
| ATP5I | 0.686275619 | 0.001396477 | 1.2437992 | 0.1209465 |
| ZDBF2 | -0.85686611 | 0.001404551 | -0.733922771 | 0.139254691 |
| E4F1 | 1.099011201 | 0.001417728 | 1.30284701 | 0.04488011 |
| CTSF | 0.751297889 | 0.001418242 | 1.29306946 | 0.045104264 |
| STK19 | 0.743001259 | 0.001418242 | 0.854560684 | 0.060205381 |
| DNLZ | 0.969996695 | 0.001432472 | 1.016708401 | 0.069614241 |
| CCS | 0.798433646 | 0.001469459 | 1.306293699 | 0.04039858 |
| CCDC42B | 0.510737894 | 0.001481347 | 0.527372367 | 0.057252434 |
| NAT9 | 0.618784179 | 0.001499615 | 0.594975142 | 0.111655625 |
| FASTK | 0.578011839 | 0.001502504 | 0.604949342 | 0.076746194 |
| CRYBG3 | -0.651639521 | 0.001502504 | -0.503006224 | 0.148282834 |
| PRKD3 | -0.504787518 | 0.001514216 | -0.534822533 | 0.122354842 |
| ZNF468 | -0.746664338 | 0.001557965 | -0.5598266 | 0.124931838 |
| CECR5 | 0.641726239 | 0.00158642 | 0.747222108 | 0.100032277 |
| CD320 | 0.741380353 | 0.001640313 | 1.336343507 | 0.032498544 |
| HNRNPH1 | -0.524034519 | 0.001642118 | -0.613385444 | 0.109402301 |
| TCEB2 | 0.646767595 | 0.001703218 | 0.791796147 | 0.11353046 |
| GJB6 | -0.632434659 | 0.001728734 | -1.10726499 | 0.129777291 |
| LOC642776 | -0.649721862 | 0.001728734 | -1.049355109 | 0.133717303 |
| RFTN2 | -0.608663936 | 0.001728734 | -0.638529182 | 0.145040987 |
| FKBP8 | 0.616255535 | 0.001740418 | 0.919098961 | 0.045347239 |
| PTGES2 | 0.657518386 | 0.00174856 | 0.885976913 | 0.056413017 |
| ETFB | 0.904905093 | 0.001774396 | 1.976878233 | 0.04039858 |
| ANP32E | -0.533028992 | 0.001774396 | -0.577060029 | 0.078664563 |
| ANKRD39 | 0.641633612 | 0.001774396 | 0.625916679 | 0.098921463 |
| C11orf83 | 0.91497157 | 0.001777837 | 1.295008647 | 0.098921463 |
| EIF3D | 0.541328753 | 0.001803425 | 1.23554148 | 0.102574048 |
| MIF4GD | 0.55069297 | 0.001817134 | 0.624718136 | 0.1319451 |
| SHPRH | -0.852105449 | 0.001831524 | -0.646402709 | 0.138991238 |
| MRPS21 | 0.672774502 | 0.001840397 | 1.196464107 | 0.149535243 |
| U2SURP | -0.610132828 | 0.001870617 | -0.558927598 | 0.119055748 |
| GTF2E1 | -0.520071821 | 0.001874621 | -0.628535324 | 0.064412191 |
| LOXL1-AS1 | -1.275105464 | 0.001879324 | -0.891860696 | 0.052832389 |
| ZNF252P-AS1 | -0.59324854 | 0.001891354 | -0.644034679 | 0.090508148 |
| REG3G | 0.876424717 | 0.001891354 | 0.837752391 | 0.098921463 |
| ZNF652 | -0.742774818 | 0.001909899 | -0.639379453 | 0.127676971 |
| RBMXL1 | -0.818169196 | 0.00195758 | -0.784719847 | 0.127629342 |
| POLR1C | 0.766606594 | 0.001979476 | 0.778063709 | 0.098408974 |
| MGMT | 1.087654376 | 0.001993441 | 1.380539347 | 0.046500249 |
| LOC100287497 | -1.02470855 | 0.001993441 | -0.901397066 | 0.098921463 |
| ZCCHC17 | 0.546197974 | 0.002029077 | 0.982403187 | 0.071989525 |
| MSRB2 | 0.540800619 | 0.002074153 | 0.673168887 | 0.145396699 |
| ARHGAP10 | 0.502153392 | 0.002075596 | 0.582155173 | 0.122869683 |
| S100A1 | 1.439940678 | 0.002075596 | 1.927098024 | 0.04039858 |
| CUEDC2 | 0.65219425 | 0.002075596 | 1.514314679 | 0.072804471 |
| COMMD9 | 0.617695403 | 0.002121961 | 1.379423382 | 0.071039549 |
| PRRX2 | 0.521406611 | 0.002135278 | 0.500372553 | 0.083505763 |
| PRIMPOL | -0.754298167 | 0.002140021 | -0.625668487 | 0.135093572 |
| BRI3 | 0.601620346 | 0.002140021 | 0.88252954 | 0.142120025 |
| EIF4EBP1 | 0.89736271 | 0.002142343 | 1.358839851 | 0.05261046 |
| TIGD5 | 0.686549807 | 0.002157222 | 0.738377903 | 0.056166142 |
| PLEKHM3 | -0.525803643 | 0.002196233 | -0.602816309 | 0.098311119 |
| SPTY2D1 | -0.63249199 | 0.002228544 | -0.519013529 | 0.060205381 |
| FLJ31306 | -0.636986046 | 0.002271651 | -0.605599364 | 0.139573732 |
| SPATA33 | 0.719059884 | 0.002290914 | 0.765171833 | 0.073882457 |
| SELM | 1.013510981 | 0.002307983 | 1.25859015 | 0.060764865 |
| GADD45GIP1 | 0.751202107 | 0.002324544 | 0.997461409 | 0.057819243 |
| KIF3A | -0.502586711 | 0.002324544 | -0.643577696 | 0.133984761 |
| 7-Sep | -0.557007256 | 0.002367624 | -0.587706433 | 0.138842562 |
| FIBP | 0.809796963 | 0.002429811 | 1.145408716 | 0.099179738 |
| IMPDH2 | 0.775437797 | 0.002549823 | 1.656244637 | 0.073227545 |
| TRMU | 0.612072409 | 0.002556136 | 0.815522414 | 0.056482781 |
| KLHL2 | -0.573155317 | 0.002556136 | -0.583872202 | 0.066640616 |
| SAMD12 | -0.559521107 | 0.002556136 | -0.761595518 | 0.047181737 |
| NENF | 0.736253951 | 0.002558035 | 0.99597078 | 0.109402301 |
| DPP3 | 0.74704195 | 0.002603721 | 0.86118834 | 0.1274872 |
| TNRC6B | -0.708692859 | 0.002603849 | -0.661924751 | 0.120421316 |
| VPS51 | 1.122998836 | 0.002603849 | 1.155901978 | 0.135835436 |
| LOC100506014 | 0.683983587 | 0.002603849 | 0.828803753 | 0.072330898 |
| CLCN7 | 0.600001032 | 0.002603849 | 0.661672231 | 0.093438185 |
| ASNA1 | 0.723361943 | 0.002615395 | 1.250334733 | 0.041586502 |
| HOOK3 | -0.536735885 | 0.002623018 | -0.523308002 | 0.133945434 |
| RP2 | -0.640876339 | 0.002629729 | -0.69904217 | 0.096004993 |
| NUDT16L1 | 0.615383416 | 0.00263755 | 1.423328994 | 0.056166142 |
| NDUFB10 | 0.57977237 | 0.002654645 | 1.347430751 | 0.04039858 |
| TMX3 | -0.540710947 | 0.002675979 | -0.714892641 | 0.110398592 |
| DYNLL1-AS1 | 0.506962699 | 0.002675979 | 0.632078139 | 0.080503837 |
| SERTAD1 | 0.643103669 | 0.002679406 | 0.802780764 | 0.068527186 |
| PPP2R4 | 0.502435228 | 0.002683142 | 0.838319162 | 0.128229202 |
| C19orf10 | 0.657669959 | 0.002690715 | 0.887699582 | 0.096893421 |
| XAB2 | 0.588923259 | 0.002692177 | 1.054225289 | 0.04039858 |
| SEC22B | -0.602185619 | 0.002737162 | -0.702360827 | 0.073227545 |
| PKDCC | 0.850076455 | 0.002739106 | 0.868104458 | 0.056413017 |
| AURKAIP1 | 0.509267456 | 0.002765453 | 1.116930738 | 0.056166142 |
| DUSP23 | 0.825249821 | 0.002794415 | 1.516975816 | 0.07922176 |
| STARD9 | -0.830847066 | 0.002800301 | -0.711236636 | 0.144099562 |
| LOC645984 | -0.951669546 | 0.002816453 | -1.014636383 | 0.083583019 |
| MRPL15 | 0.576771436 | 0.002831355 | 1.427904787 | 0.062878082 |
| ACTR1B | 0.507499095 | 0.002839013 | 0.88759308 | 0.041586502 |
| GNE | -1.166318635 | 0.002858657 | -1.172569786 | 0.104966935 |
| GYPC | 1.17380529 | 0.002896038 | 1.344409133 | 0.098347728 |
| PPP4C | 0.83549555 | 0.002919609 | 1.284371427 | 0.047482879 |
| SNRNP25 | 0.67579175 | 0.002930565 | 1.345595773 | 0.107716807 |
| RP3-368A4.6 | -0.887374335 | 0.002930565 | -1.106956902 | 0.054628716 |
| RBM25 | -0.658659617 | 0.002959535 | -0.715195573 | 0.098408974 |
| KAT5 | 0.616991221 | 0.003005307 | 1.097218911 | 0.035072496 |
| ACBD6 | 0.809009349 | 0.003031876 | 0.651052949 | 0.108562044 |
| LOC285812 | -0.558557671 | 0.003042398 | -0.829189816 | 0.111876096 |
| MYL6B | 0.865554776 | 0.003076608 | 1.727644833 | 0.073533577 |
| MRPS9 | 0.697406535 | 0.003085192 | 0.913283873 | 0.128212671 |
| MRPS34 | 0.834929983 | 0.003130709 | 1.132213001 | 0.077321195 |
| NMRAL1 | 0.572153566 | 0.003148642 | 0.934998938 | 0.097859395 |
| OR51B4 | -0.548452432 | 0.003198659 | -0.532998728 | 0.103649937 |
| KLRAP1 | -0.752367926 | 0.003235125 | -1.418631204 | 0.094427732 |
| TNFRSF12A | 0.861580348 | 0.003280269 | 0.956475422 | 0.074861744 |
| ATP11B | -0.623930675 | 0.003406945 | -0.631336179 | 0.108920141 |
| CDC20 | 0.57268493 | 0.003427239 | 0.552436092 | 0.129562344 |
| EMC8 | 0.504124041 | 0.003462307 | 0.756018896 | 0.109519047 |
| ABHD12 | 0.585332292 | 0.003471997 | 0.794810642 | 0.047181737 |
| GTF2H4 | 0.77181272 | 0.003548383 | 1.268962744 | 0.028818306 |
| CHMP2A | 0.581002929 | 0.003558878 | 1.076957457 | 0.120547546 |
| REL | -0.504397205 | 0.003638156 | -0.720658471 | 0.076746194 |
| SHARPIN | 0.758383305 | 0.003651482 | 0.831951538 | 0.142268964 |
| LMBRD1 | -0.567275981 | 0.003651482 | -0.568131018 | 0.129556062 |
| MIEN1 | 0.701973461 | 0.003664309 | 1.132374562 | 0.131790001 |
| NINJ1 | 0.842576543 | 0.003726242 | 0.998383144 | 0.101786208 |
| PEMT | 0.790102656 | 0.003739159 | 0.846696342 | 0.108920141 |
| LOC101929787 | -1.013370419 | 0.003762429 | -1.191032702 | 0.035783122 |
| STIM2 | -0.58945103 | 0.003763458 | -0.723068113 | 0.071522614 |
| HEATR5A | -0.538613181 | 0.003793759 | -0.53221815 | 0.128890533 |
| FIS1 | 0.755411706 | 0.003840238 | 1.496492529 | 0.091239717 |
| HEATR5B | -0.596598074 | 0.003842541 | -0.830366469 | 0.028818306 |
| ASL | 1.337598519 | 0.00390346 | 2.107151671 | 0.035072496 |
| PNKP | 1.094916804 | 0.003912753 | 1.154926173 | 0.104731994 |
| HEXIM2 | 0.856094876 | 0.003912753 | 0.824086261 | 0.116613938 |
| WDR18 | 0.702540796 | 0.00393127 | 0.635248333 | 0.146613461 |
| FBXW9 | 0.610519161 | 0.004083586 | 0.713205341 | 0.056413017 |
| WDR45 | 0.750029459 | 0.004083586 | 1.225940494 | 0.041883585 |
| MAP4K4 | -0.652082611 | 0.004110588 | -0.8503521 | 0.04039858 |
| ANAPC15 | 0.627801903 | 0.004127278 | 1.05569528 | 0.075625181 |
| MLST8 | 0.652983908 | 0.004157481 | 1.257169291 | 0.028818306 |
| DGAT1 | 0.704274922 | 0.00415848 | 0.732545514 | 0.11627447 |
| FAM154B | -0.804318362 | 0.004168267 | -0.733451668 | 0.123712395 |
| UNC5D | -0.76533109 | 0.004169273 | -0.846047469 | 0.128735803 |
| RASSF7 | 0.698549374 | 0.004256097 | 0.762580298 | 0.124113253 |
| GNPTG | 0.676412839 | 0.004268343 | 1.20576716 | 0.096004993 |
| RPL29 | 0.624223486 | 0.004328614 | 1.408094398 | 0.131582639 |
| DHRS13 | 0.864827583 | 0.004342868 | 0.921210057 | 0.080639035 |
| CLPP | 0.741461074 | 0.004365995 | 1.586402749 | 0.042423702 |
| RP11-488L18.10 | -0.862588081 | 0.004366899 | -1.07226201 | 0.109559764 |
| NRAS | -0.631228113 | 0.004378744 | -0.728121293 | 0.104731994 |
| AX747507 | -1.136315565 | 0.004400583 | -0.749096072 | 0.136735794 |
| ECSIT | 0.725668992 | 0.004400583 | 1.236816638 | 0.076094854 |
| RNPEPL1 | 0.563169143 | 0.00440288 | 0.769140319 | 0.123799632 |
| TIMM10 | 0.805258905 | 0.004416817 | 0.975328258 | 0.062878082 |
| TAF5 | -0.59027805 | 0.004416817 | -0.563317471 | 0.094775391 |
| SCAND1 | 0.690789736 | 0.004451695 | 0.896168678 | 0.105395214 |
| SLC1A5 | 0.673229284 | 0.004462467 | 0.874314494 | 0.035783122 |
| CORO1B | 0.560650941 | 0.004462467 | 0.518648284 | 0.128735803 |
| ARRDC3 | -0.764321599 | 0.004475692 | -0.836721726 | 0.119245634 |
| C6orf165 | 0.604087194 | 0.00459589 | 0.539692854 | 0.138991238 |
| TAS2R13 | -0.500587448 | 0.004617569 | -0.648919716 | 0.135835436 |
| TMEM141 | 0.788205786 | 0.004639886 | 1.444373556 | 0.042423702 |
| FDX1L | 0.686410117 | 0.004729404 | 1.440436136 | 0.04039858 |
| TSSC4 | 0.584314081 | 0.004884771 | 1.108655153 | 0.073227545 |
| LOC285147 | -0.844037596 | 0.004903868 | -1.042149022 | 0.055162758 |
| ERV3-1 | -0.679510462 | 0.004952988 | -0.787392252 | 0.128207025 |
| ZSWIM6 | -0.52705473 | 0.004991386 | -0.807099408 | 0.071617502 |
| EIF2B3 | 0.603159742 | 0.005153791 | 0.659822976 | 0.109559764 |
| MCRS1 | 0.567937869 | 0.005180589 | 1.149896747 | 0.066865445 |
| KANSL1 | -0.802661633 | 0.005221829 | -0.8129386 | 0.111512195 |
| RP5-930J4.4 | -0.618239361 | 0.005247576 | -0.598166136 | 0.125619574 |
| ECI1 | 0.739469425 | 0.005272627 | 0.865181778 | 0.131783285 |
| RP11-774O3.3 | -0.560255979 | 0.005272627 | -0.650102419 | 0.097001299 |
| SPA17 | 0.877774902 | 0.005298867 | 0.945324117 | 0.089449667 |
| EDEM2 | 0.516608898 | 0.005299644 | 1.074814702 | 0.107455195 |
| APRT | 0.584144164 | 0.005395655 | 1.041677484 | 0.104602607 |
| NDUFV3 | 0.590132295 | 0.005441562 | 1.086470451 | 0.074858347 |
| CCDC22 | 0.660668681 | 0.005547044 | 0.808779404 | 0.109559764 |
| TSSC1 | 0.623470354 | 0.005637482 | 0.736229853 | 0.146613461 |
| LRIF1 | -0.787284753 | 0.005735229 | -0.816322422 | 0.109519047 |
| LAMTOR2 | 0.781643564 | 0.005903382 | 1.441364453 | 0.035783122 |
| ASPSCR1 | 0.633559523 | 0.00599136 | 0.905622474 | 0.04696954 |
| ENDOG | 0.883153296 | 0.00620945 | 1.475049972 | 0.056166142 |
| SPSB2 | 0.659436615 | 0.006251445 | 0.791150158 | 0.08167561 |
| DDAH2 | 0.569488374 | 0.006279529 | 0.923704996 | 0.10465161 |
| C16orf91 | 0.615992948 | 0.006354609 | 0.841053853 | 0.130038342 |
| C1orf53 | 0.594647588 | 0.006414108 | 0.762364207 | 0.093696607 |
| LOC389332 | 1.3083782 | 0.006459068 | 1.975183216 | 0.028818306 |
| PSMC4 | 0.522497337 | 0.006459068 | 0.976107578 | 0.146532315 |
| ERI3 | 0.751273765 | 0.006459068 | 1.555171688 | 0.035072496 |
| FAM50A | 0.719266896 | 0.006576643 | 1.071880377 | 0.083611973 |
| DDX41 | 0.782654042 | 0.006580274 | 1.204929058 | 0.143654477 |
| GUK1 | 0.653815718 | 0.006617185 | 1.746354558 | 0.056413017 |
| NSMCE2 | 0.732308659 | 0.006692337 | 0.881246704 | 0.1209465 |
| SHISA2 | -1.066510604 | 0.006692337 | -0.929520724 | 0.127851554 |
| PUF60 | 0.688654437 | 0.006692337 | 1.00341976 | 0.078548363 |
| KIF22 | 0.547503679 | 0.006821721 | 0.632065956 | 0.123524028 |
| SLC38A9 | -0.637456699 | 0.006904615 | -0.685824602 | 0.135874269 |
| RNF181 | 0.697316305 | 0.006932162 | 1.460109156 | 0.07995039 |
| COA4 | 0.626774806 | 0.006932162 | 1.098944096 | 0.098921463 |
| F3 | -0.964144189 | 0.006935769 | -0.749181124 | 0.118318019 |
| ANP32A-IT1 | -0.810149969 | 0.006969175 | -0.968275916 | 0.075102135 |
| LOC100996579 | -0.780108673 | 0.007074717 | -1.027277507 | 0.071039549 |
| TMEM53 | 0.608498099 | 0.007119889 | 0.719071498 | 0.112392346 |
| GADD45B | 0.723271023 | 0.007123679 | 0.756679844 | 0.116613938 |
| GNB2 | 0.622266039 | 0.007174875 | 1.148660758 | 0.028818306 |
| ETHE1 | 0.855397061 | 0.007204685 | 1.055997596 | 0.109342669 |
| AK090844 | -0.594621119 | 0.007215887 | -1.001606918 | 0.083505763 |
| GCA | -0.636876679 | 0.007278867 | -0.587432623 | 0.111650845 |
| TKT | 0.595261264 | 0.007278867 | 1.1070611 | 0.04696954 |
| LACTB2 | 0.750918908 | 0.007282279 | 1.100549453 | 0.061030101 |
| SMNDC1 | -0.52017077 | 0.007288857 | -0.546030807 | 0.102093113 |
| BAX | 0.772542313 | 0.007314806 | 1.08575535 | 0.128253438 |
| GPR171 | 1.022516984 | 0.007346026 | -1.47747269 | 0.078897913 |
| C9orf114 | 0.524610899 | 0.007516345 | 0.828137431 | 0.086957159 |
| FBXO25 | 0.56758209 | 0.008067715 | 1.034906916 | 0.079212305 |
| NOTCH2NL | -0.604733036 | 0.008076582 | -1.183607733 | 0.045347239 |
| NDUFA13 | 0.537701821 | 0.008087081 | 1.207955416 | 0.121904161 |
| LINC00934 | -0.526795388 | 0.00809195 | -0.660202736 | 0.106596908 |
| RPL26L1 | 0.639896589 | 0.008235726 | 1.059338311 | 0.137554116 |
| MRPL57 | 0.517533603 | 0.008287484 | 0.732967793 | 0.122354842 |
| CHMP6 | 0.578106255 | 0.008318027 | 0.836108127 | 0.096893421 |
| PCBP4 | 0.58955812 | 0.008321136 | 0.660879948 | 0.055194593 |
| AC005606.14 | 0.690199888 | 0.00839121 | 1.135029853 | 0.052832389 |
| LONP1 | 0.861583284 | 0.008396148 | 1.209005866 | 0.045356538 |
| RNF44 | -0.553875422 | 0.00846272 | -0.636526871 | 0.131783285 |
| CHD1 | -0.527354968 | 0.008673013 | -0.855930649 | 0.035641138 |
| GAS2L1 | 0.612957121 | 0.008705684 | 0.868668207 | 0.097859395 |
| C4orf46 | -0.604202125 | 0.008745059 | -0.96741474 | 0.056166142 |
| LOC100287015 | -0.548470776 | 0.008767177 | -0.541302207 | 0.108746261 |
| BLVRB | 0.640236548 | 0.008795682 | 1.483706777 | 0.091054193 |
| NAPRT | 0.815812778 | 0.008816939 | 1.374730296 | 0.052832389 |
| DNPH1 | 0.899162073 | 0.008945722 | 1.598933796 | 0.05261046 |
| IL17RB | 0.942449484 | 0.009026428 | 1.425858831 | 0.086585124 |
| EGFL7 | 0.650442824 | 0.009173409 | 0.817590246 | 0.10465161 |
| PSMD8 | 0.585855509 | 0.009228825 | 1.586059353 | 0.088827513 |
| FAM83G | 0.549239901 | 0.009257133 | 0.660708618 | 0.061898706 |
| KDM7A | -0.527385279 | 0.009262664 | -0.778599944 | 0.047701645 |
| SMC6 | -0.506752272 | 0.009391286 | -0.973526451 | 0.04039858 |
| EIF6 | 0.543761896 | 0.009404438 | 1.043941598 | 0.0937583 |
| ENKD1 | 0.573774632 | 0.009588635 | 1.198822516 | 0.061791766 |
| BABAM1 | 0.619403185 | 0.009716421 | 1.424827728 | 0.04039858 |
| SMIM19 | 0.541859772 | 0.010138678 | 1.111842618 | 0.105833857 |
| ISOC2 | 0.833894183 | 0.010225789 | 1.810008544 | 0.034089497 |
| LY9 | 0.627240392 | 0.010230987 | -0.71667435 | 0.143353676 |
| TEKT2 | 0.673261829 | 0.01024193 | 0.716160469 | 0.111512195 |
| TEX264 | 0.612805681 | 0.010265641 | 1.458864433 | 0.04039858 |
| TMED1 | 0.629285991 | 0.010272523 | 1.253747712 | 0.086957159 |
| PEAR1 | -0.768034294 | 0.010335079 | -0.762856544 | 0.081730013 |
| POR | 0.787301449 | 0.010553316 | 1.046613147 | 0.074940056 |
| NIT2 | 0.623254563 | 0.010586921 | 1.238021421 | 0.123258621 |
| IQGAP1 | -0.549067171 | 0.010717309 | -0.615282456 | 0.128229202 |
| PFKFB3 | -0.951562352 | 0.010725739 | -0.813309892 | 0.07922176 |
| CCDC146 | 0.874257262 | 0.010876522 | 1.073263462 | 0.096004993 |
| C7orf55 | 0.675740808 | 0.010935456 | 1.883720753 | 0.04039858 |
| PITPNM1 | 0.553467405 | 0.011078292 | 0.71458818 | 0.083170241 |
| FAM3A | 0.582176661 | 0.011135353 | 0.861400453 | 0.046705259 |
| NAA38 | 0.72308089 | 0.011221133 | 1.095064816 | 0.104673369 |
| TOPORS | -0.60646475 | 0.011261946 | -0.677059864 | 0.142120025 |
| RP11-119F7.5 | -0.568076631 | 0.011444188 | -0.659291226 | 0.149898727 |
| UTP15 | -0.521995958 | 0.011747557 | -0.697775229 | 0.114155521 |
| CDK5 | 0.762853306 | 0.011804815 | 1.346647623 | 0.045347239 |
| KMT2E-AS1 | 0.663143598 | 0.012108819 | 0.846570374 | 0.109519047 |
| PALM | 0.639722054 | 0.012229053 | 1.223406624 | 0.033044365 |
| LOC100506498 | 1.168901196 | 0.012292307 | 0.903776659 | 0.149886741 |
| METRN | 0.524829287 | 0.012429036 | 0.723212449 | 0.083402492 |
| RPP25L | 0.521896434 | 0.012552802 | 0.837881947 | 0.074861744 |
| GVINP1 | 0.600701309 | 0.013337159 | -1.015478604 | 0.083434503 |
| NDUFB7 | 0.613701036 | 0.013379158 | 1.110429229 | 0.091250159 |
| PKN1 | 0.586204421 | 0.013480236 | 0.806564889 | 0.132363382 |
| RP11-1024P17.1 | -0.528702353 | 0.013544898 | -1.11871098 | 0.121272894 |
| BAZ2B | -0.554932829 | 0.014548942 | -0.594098527 | 0.139172713 |
| CSDC2 | 0.928441629 | 0.014610027 | 1.334571629 | 0.028818306 |
| PLEKHA4 | 0.522577998 | 0.014665999 | 0.599674988 | 0.123762851 |
| LOC100506119 | 0.882834334 | 0.014927442 | 1.120045814 | 0.08852713 |
| ASMTL | 0.624266051 | 0.014962159 | 0.801335804 | 0.101132497 |
| 9-Mar | 0.690582427 | 0.014980467 | 1.13953782 | 0.046500249 |
| LOC644656 | 0.845631835 | 0.015185627 | 1.057633791 | 0.1319451 |
| RPS5 | 0.550913192 | 0.015192336 | 1.469651569 | 0.098921463 |
| RNF186 | 1.081857949 | 0.015199103 | 1.728944893 | 0.044057138 |
| CHID1 | 0.534146502 | 0.015288912 | 1.605569538 | 0.05261046 |
| SCRN2 | 0.574948044 | 0.01557006 | 0.684357796 | 0.064855701 |
| TRAPPC12 | 0.754193123 | 0.015800607 | 1.102516456 | 0.109402301 |
| ALG14 | 0.706958904 | 0.01589124 | 0.815037498 | 0.103649937 |
| CLASRP | 0.653793954 | 0.015922594 | 1.231324149 | 0.128735803 |
| COMTD1 | 0.652312485 | 0.016013542 | 1.171212454 | 0.080606111 |
| ETFA | 0.649148242 | 0.016139263 | 1.167984949 | 0.085657449 |
| MVB12A | 0.650022573 | 0.016249039 | 0.920852233 | 0.104607039 |
| PHYHIP | 0.677390075 | 0.016580914 | 0.827946821 | 0.111116633 |
| ARL6IP4 | 0.647129699 | 0.016765089 | 1.406061902 | 0.042423702 |
| MRPL46 | 0.603696989 | 0.016885455 | 1.294258518 | 0.118722417 |
| PFDN2 | 0.647993584 | 0.01707919 | 1.167537096 | 0.072330898 |
| KIAA1024 | -0.608048351 | 0.017265975 | -0.691575493 | 0.133382837 |
| AP2S1 | 0.535768148 | 0.017316684 | 1.054277913 | 0.042423702 |
| MVP | 0.698028544 | 0.017357891 | 1.181337434 | 0.079804705 |
| NPR1 | 0.532791218 | 0.017433752 | 0.845162762 | 0.10465161 |
| MRPL14 | 0.671942407 | 0.01766311 | 0.951469583 | 0.098921463 |
| FAM96B | 0.628364035 | 0.017766517 | 1.325792682 | 0.078899238 |
| MRPS26 | 0.576798622 | 0.017766517 | 1.17393168 | 0.062878082 |
| TRAF3IP3 | 0.567310766 | 0.018027132 | -0.764668887 | 0.123958077 |
| ACBD4 | 0.58413275 | 0.018488255 | 1.062550762 | 0.028818306 |
| ZNF2 | -0.611207569 | 0.018492966 | -0.598334812 | 0.118312396 |
| SNRPA | 0.640698267 | 0.018763806 | 0.847429401 | 0.130038342 |
| IGFBP6 | 0.930593906 | 0.018797927 | 1.029283412 | 0.144947668 |
| SH3TC1 | 0.600297576 | 0.01929228 | 0.852091153 | 0.054559791 |
| RAMP2 | 0.514410156 | 0.019368455 | 1.163764124 | 0.064074172 |
| KIAA1462 | -0.55280509 | 0.01965659 | -0.665514849 | 0.113804723 |
| LIME1 | 0.889740936 | 0.020343484 | 1.155226427 | 0.062878082 |
| MYL9 | 0.820742693 | 0.020704742 | 1.278722776 | 0.076746194 |
| ELMO3 | 0.72122005 | 0.020724237 | 1.447137634 | 0.035072496 |
| NEURL4 | 0.649769753 | 0.02081816 | 0.819112528 | 0.068527186 |
| YIF1A | 0.524578791 | 0.021039237 | 1.135851533 | 0.045301246 |
| SCT | 0.600340629 | 0.021139962 | 0.63283236 | 0.127629342 |
| MTFP1 | 0.555518572 | 0.021139962 | 0.796129904 | 0.118500256 |
| RABAC1 | 0.574230942 | 0.02152804 | 1.432676049 | 0.034020192 |
| GALK1 | 0.511532573 | 0.021678782 | 0.892557236 | 0.067382629 |
| RP4-614O4.12 | -0.515477896 | 0.021904776 | -1.020137627 | 0.074858347 |
| GPS1 | 0.579863726 | 0.021940559 | 1.595649164 | 0.028863249 |
| SNORA21 | -0.682670228 | 0.022068021 | -0.858054403 | 0.096567498 |
| ZFYVE19 | 0.626358171 | 0.022120141 | 1.192947336 | 0.082959256 |
| BCKDK | 0.583890883 | 0.022219226 | 1.125350921 | 0.083505763 |
| IQCD | 0.509927028 | 0.022882171 | 0.638365234 | 0.124444696 |
| MRPL13 | 0.593559237 | 0.023074069 | 1.039285251 | 0.062879486 |
| DCXR | 0.971916281 | 0.023121825 | 1.577635173 | 0.065264277 |
| ZMYND19 | 0.554823853 | 0.023166363 | 1.11326654 | 0.073640007 |
| GDF15 | 0.530674697 | 0.023249463 | 0.813385496 | 0.06785078 |
| CLTB | 0.564955416 | 0.023297405 | 1.200529116 | 0.043736987 |
| GLB1L2 | 0.855710575 | 0.023343819 | 1.560602238 | 0.032498544 |
| SPR | 0.621137352 | 0.023343819 | 1.623738856 | 0.04039858 |
| CPOX | -0.51022112 | 0.023450367 | -0.803277291 | 0.097578422 |
| TTLL1 | 0.524505404 | 0.023554911 | 0.785496487 | 0.113373096 |
| MIS12 | -0.537411084 | 0.023554911 | -0.790577389 | 0.074940056 |
| GCAT | 0.649037231 | 0.023559966 | 0.925051278 | 0.098921463 |
| ASCC2 | 0.553370957 | 0.023745512 | 1.195018196 | 0.072014955 |
| ZNF511 | 0.668151442 | 0.023972936 | 1.203836747 | 0.079032436 |
| CPTP | 0.541422112 | 0.024097586 | 0.997283351 | 0.076094854 |
| SURF2 | 0.591458936 | 0.02441503 | 1.24173172 | 0.04039858 |
| EFCAB4A | 0.508219328 | 0.024617247 | 0.609370091 | 0.0937583 |
| SLC22A18AS | 0.968558214 | 0.02608646 | 1.115558926 | 0.085090795 |
| UQCR10 | 0.527321111 | 0.026421429 | 1.391941602 | 0.052832389 |
| MRPL12 | 0.679495293 | 0.026629188 | 1.515116922 | 0.028818306 |
| TST | 0.868830156 | 0.027228306 | 1.308740176 | 0.120261375 |
| AKR7A3 | 0.799641587 | 0.027380139 | 1.794729926 | 0.035072496 |
| PARD3-AS1 | 0.639359897 | 0.027887651 | 0.830003218 | 0.098921463 |
| PRKAR2B | -0.837404513 | 0.02817629 | -0.792794512 | 0.138991238 |
| ZNF593 | 0.768325175 | 0.028233734 | 1.950603427 | 0.035731451 |
| WDR46 | 0.523490846 | 0.02825206 | 0.924921151 | 0.068527186 |
| CCDC51 | 0.645469582 | 0.02913065 | 0.92437098 | 0.128229202 |
| SLC39A12 | -0.536867484 | 0.029433715 | -1.022461622 | 0.130881194 |
| PACSIN3 | 0.524440394 | 0.029539932 | 0.982194627 | 0.044552566 |
| TAGLN | 0.773127653 | 0.029545976 | 1.108721582 | 0.115150183 |
| SLC5A2 | 1.102181773 | 0.030314159 | 1.423453807 | 0.051223932 |
| TUBG1 | 0.763624084 | 0.03082698 | 1.229467403 | 0.068527186 |
| CXXC1 | 0.554164934 | 0.03088261 | 0.734412582 | 0.104731994 |
| LOC101927752 | 0.536096684 | 0.030962805 | 1.052900842 | 0.08173383 |
| SH3GL1 | 0.615891763 | 0.031009059 | 1.02458794 | 0.103649937 |
| ADIRF | 0.651978916 | 0.031274481 | 1.51958375 | 0.096555094 |
| SIGIRR | 0.622556764 | 0.031507962 | 1.383912196 | 0.081832419 |
| ISG15 | 1.02527823 | 0.032351655 | 1.093466071 | 0.147385481 |
| CCDC107 | 0.608029873 | 0.032427428 | 1.148026451 | 0.131790001 |
| MEIS2 | -0.528945613 | 0.03260193 | -0.637499011 | 0.116001926 |
| MTCL1 | -0.854372148 | 0.032955363 | -1.103961266 | 0.10330978 |
| BOK | 0.700959445 | 0.033367422 | 1.120540071 | 0.0937583 |
| GSTZ1 | 0.623890207 | 0.033393338 | 1.34577347 | 0.062489247 |
| PRF1 | 0.625906435 | 0.033657952 | -1.007686793 | 0.118198615 |
| C12orf10 | 0.510267667 | 0.033956972 | 1.102955908 | 0.07890755 |
| SLC39A5 | 1.001172805 | 0.034330832 | 1.794352838 | 0.035072496 |
| PCSK1N | 0.837867416 | 0.035510164 | 1.436260369 | 0.113373096 |
| OSBPL3 | -0.627924713 | 0.036395861 | -0.893347247 | 0.047645179 |
| ACOT13 | 0.729863623 | 0.036432566 | 1.785163596 | 0.083505763 |
| GAMT | 0.524374863 | 0.036569328 | 0.767879427 | 0.139254691 |
| ECHDC3 | 0.645553661 | 0.036637573 | 1.544348744 | 0.047181737 |
| BLOC1S1 | 0.60148041 | 0.037505834 | 2.079807158 | 0.056568745 |
| NUDT2 | 0.522805001 | 0.037556548 | 1.040628837 | 0.127209841 |
| C19orf70 | 0.534542219 | 0.038753729 | 1.038027004 | 0.109559764 |
| MED11 | 0.633470527 | 0.038781975 | 1.485489058 | 0.077487208 |
| FCRLB | 0.642587536 | 0.038972853 | 0.908271144 | 0.096004993 |
| LY75 | -0.970362008 | 0.039301037 | -2.106104489 | 0.028818306 |
| B9D1 | 0.561683387 | 0.040243917 | 0.953502491 | 0.052832389 |
| CDK18 | 0.798981174 | 0.040407654 | 1.291329444 | 0.056413017 |
| NAGPA | 0.525526817 | 0.041203991 | 0.882724133 | 0.083420924 |
| EOMES | 0.957913324 | 0.041744595 | -1.477918922 | 0.068527186 |
| RNF175 | 0.549981358 | 0.04185844 | -0.92368449 | 0.141403941 |
| RP11-38P22.2 | -0.64358047 | 0.042268808 | -1.057879083 | 0.0914643 |
| SPHK2 | 0.683387531 | 0.04310704 | 0.886505624 | 0.083596597 |
| CYP27A1 | 0.603094296 | 0.043839893 | 1.326063444 | 0.035731451 |
| CTC-471J1.2 | -0.594123919 | 0.04452746 | -0.717736074 | 0.11724761 |
| HYAL2 | 0.527112664 | 0.045802942 | 1.490742371 | 0.04039858 |
| DECR2 | 0.784084346 | 0.046485181 | 0.971448278 | 0.140178096 |
| BCHE | 0.814778439 | 0.046519875 | 1.091295579 | 0.109559764 |
| LOC100505942 | 0.568425229 | 0.046637695 | 0.877625947 | 0.131790001 |
| PCBD1 | 0.557540037 | 0.047497771 | 1.700101004 | 0.044057138 |
| FUOM | 0.752077041 | 0.047534718 | 1.379620262 | 0.062878082 |
| GLYCTK | 0.612067408 | 0.047857284 | 0.960228987 | 0.105329366 |
| FOXRED1 | 0.580496957 | 0.048339816 | 1.152415124 | 0.086877481 |
| SLC30A2 | 0.652073307 | 0.049410407 | 0.877627856 | 0.096893421 |
| FTCD | 0.777562247 | 0.049410407 | 1.258225982 | 0.071522614 |
| NUPR1 | 0.568582715 | 0.049862861 | 1.270917971 | 0.102818483 |
| RBM42 | 0.705319126 | 0.050239097 | 1.295740638 | 0.056482781 |
| HPD | 1.495737168 | 0.050896799 | 2.810097698 | 0.035072496 |
| LDLRAD3 | -0.558810398 | 0.051704216 | -0.796537684 | 0.12536977 |
| RASD1 | 0.632030784 | 0.051754009 | 0.845586218 | 0.096567498 |
| FOLR3 | 0.87257862 | 0.053651345 | 1.544609971 | 0.048073161 |
| DHDH | 0.955428545 | 0.055037596 | 1.571038514 | 0.052362868 |
| SLC22A18 | 0.848988472 | 0.055855961 | 1.53687274 | 0.086555781 |
| SDSL | 0.571604069 | 0.058206911 | 1.527770069 | 0.042423702 |
| THBD | -0.560021333 | 0.060367409 | -0.678992722 | 0.104984677 |
| HAAO | 0.717119368 | 0.06139825 | 1.386123578 | 0.04039858 |
| AOX1 | 0.76628883 | 0.064891037 | 0.853802927 | 0.127764966 |
| PROC | 0.714465193 | 0.064922288 | 0.999167129 | 0.110539956 |
| ANKRD2 | 0.800810787 | 0.065429983 | 1.13544438 | 0.144186293 |
| GHDC | 0.501437675 | 0.066796449 | 0.951625042 | 0.114043547 |
| RP11-158G18.1 | -0.520504267 | 0.068261275 | -1.289625486 | 0.079804705 |
| CRTAM | 0.699140284 | 0.07020298 | -1.601985606 | 0.045104264 |
| PRAP1 | 1.114468034 | 0.070662917 | 1.855799759 | 0.04696954 |
| MSRB1 | 0.58750015 | 0.071626671 | 1.555919786 | 0.068377893 |
| DNAJC12 | 0.791897214 | 0.073392588 | 0.96183648 | 0.103649937 |
| TNFSF4 | -0.53159997 | 0.075102501 | -0.698824698 | 0.121408218 |
| KHK | 0.743392573 | 0.076000916 | 1.770284259 | 0.034089497 |
| RP11-44F21.5 | 1.055302992 | 0.082707383 | 1.523409159 | 0.083904484 |
| IFNG | 0.506507901 | 0.082856264 | -1.001024548 | 0.144099562 |
| PTGS1 | -0.619227586 | 0.082949886 | -0.835218553 | 0.080606111 |
| CYP27B1 | 1.160065297 | 0.083335 | 1.874195053 | 0.07518071 |
| NQO2 | 0.605687259 | 0.085227327 | 1.281809742 | 0.125277182 |
| SLC2A9 | 0.674257404 | 0.085858138 | 0.91165242 | 0.122394297 |
| ABLIM3 | 0.668694117 | 0.086199268 | 0.934609393 | 0.109070527 |
| HSD17B14 | 0.719504596 | 0.087026028 | 1.296055113 | 0.055194593 |
| AGXT | 0.615695141 | 0.087548318 | 0.876188349 | 0.104287465 |
| CRYAA | 0.806856816 | 0.088647619 | 1.588229518 | 0.04039858 |
| APOE | 0.680181127 | 0.095305642 | 0.791275193 | 0.093696607 |
| ACAA1 | 0.513942883 | 0.096541306 | 1.430864124 | 0.066687695 |
| GPR18 | 0.712527506 | 0.097876858 | -1.514107122 | 0.061933299 |
| PRSS3 | 0.593362341 | 0.099872292 | 0.894150293 | 0.098921463 |
| PI4KA | 0.502086402 | 0.100759899 | 1.069570731 | 0.11724761 |
| HPN | 0.50274528 | 0.101318541 | 0.858278947 | 0.121408218 |
| NAT8B | 0.884301448 | 0.10632931 | 1.666705344 | 0.075168133 |
| SLC3A2 | 0.557323221 | 0.106439288 | 1.613560149 | 0.036249713 |
| PRODH2 | 0.673232798 | 0.107075815 | 1.3388985 | 0.044516484 |
| TRG-AS1 | 0.635026326 | 0.107591623 | -1.41189861 | 0.055690199 |
| ACOT7 | 0.628423064 | 0.111104309 | 0.958554276 | 0.090927322 |
| RP11-297L17.2 | 0.536484511 | 0.113991781 | 1.130565531 | 0.0937583 |
| SLC17A3 | 1.015264584 | 0.116191533 | 1.296917356 | 0.148320379 |
| SMIM2-AS1 | 0.644424251 | 0.116616042 | 1.425366524 | 0.046500249 |
| GGTLC1 | 0.566660057 | 0.118813218 | 1.44132293 | 0.032610861 |
| ANG | 0.555134399 | 0.139697461 | 1.146755684 | 0.083170241 |
| RP11-700J17.2 | -0.568434164 | 0.139922121 | -0.822642374 | 0.131969282 |
| PDZK1IP1 | 0.700487157 | 0.141535146 | 1.775153127 | 0.062878082 |
| ACMSD | 0.667620573 | 0.144157409 | 0.799641756 | 0.131185891 |
| MPEG1 | 0.616284657 | 0.144945731 | -1.157899849 | 0.131185891 |
| PPP1R16A | 0.631775876 | 0.145068076 | 1.846624346 | 0.028818306 |
| SLAMF6 | 0.538954816 | 0.14861908 | -1.286283584 | 0.139232665 |

**Supplementary Table 4.** The average rank of 19 genes included in the grey60 module calculated by support vector machine - recursive feature elimination algorithm.

| STA | | AR | |
| --- | --- | --- | --- |
| Gene | AvgRank | Gene | AvgRank |
| FCRLB | 2.1 | RBBP7 | 2.2 |
| GYPC | 2.8 | GNB2 | 2.4 |
| RBBP7 | 4.3 | GADD45B | 2.7 |
| AP2S1 | 4.3 | FAM207A | 5.6 |
| EIF3D | 7 | IMPDH2 | 6.9 |
| RAMP2 | 8.7 | FCRLB | 8.3 |
| BST2 | 9.8 | FAM50A | 9.1 |
| IMPDH2 | 10.2 | GYPC | 9.7 |
| GNB2 | 10.2 | FASTK | 9.9 |
| FASTK | 10.5 | EIF3D | 10.2 |
| TAGLN | 11.9 | BST2 | 11.1 |
| DDAH2 | 12.3 | DDAH2 | 11.1 |
| FAM207A | 12.5 | KLHL2 | 13.4 |
| SERTAD1 | 13 | IFI35 | 13.7 |
| FAM50A | 13.2 | TAGLN | 13.7 |
| ISG15 | 13.4 | AP2S1 | 14.3 |
| KLHL2 | 13.8 | RAMP2 | 14.7 |
| IFI35 | 15 | ISG15 | 15.4 |
| GADD45B | 15 | SERTAD1 | 15.6 |
